# Mechanisms of Chinese Hickory Resistance to Dry Rot Disease by *Botryosphaeria dothidea*: A Comprehensive Analysis from Gene Expression to Non-Coding RNAs

**DOI:** 10.3390/plants14050793

**Published:** 2025-03-04

**Authors:** Yingshan Chen, Yuke Zhou, Jiahui Chen, Haoming Cai, Ruifeng Yang, Da Zhang, Youjun Huang

**Affiliations:** 1 National Key Laboratory for Development and Utilization of Forest Food Resources, Zhejiang A&F University, Hangzhou 311300, China; cys12072286@outlook.com (Y.C.); yuke313@outlook.com (Y.Z.); jiahuichen@stu.zafu.edu.cn (J.C.); c201006061958@163.com (H.C.); 13347164366@139.com (R.Y.); dazhang@stu.zafu.edu.cn (D.Z.); 2 Provincial Key Laboratory for Non-Wood Forest and Quality Control and Utilization of Its Products, Zhejiang A&F University, Hangzhou 311300, China

**Keywords:** *Carya cathayensis*, *Botryosphaeria dothidea*, dry rot disease, transcriptome, disease resistance pathway, lncRNA, circRNA

## Abstract

Chinese hickory (*Carya cathayensis*) is an important tree species for agriculture, but dry rot disease, caused by *Botryosphaeria dothidea*, threatens its viability. To study the interactions between the tree and the pathogen, transcriptomic sequencing was conducted on infected and healthy tissues from field-grown hickory. Differential gene expression analysis identified key defense pathways and genes activated during infection. The study also explored the roles of non-coding RNAs, such as lncRNAs and circRNAs, in the tree’s defense. The results showed that during the early and mid stages of infection, the tree defends itself through mechanisms like enhanced lignin synthesis and increased peroxidase activity. Non-coding RNAs contribute to disease resistance by reinforcing the cell wall, increasing oxidase activity, and promoting the synthesis of antibiotic-related secondary metabolites. Additionally, gene expression patterns at these stages differ significantly from those at the late stage of infection, when most disease resistance pathways are suppressed, and genes like *PR1* and *WRKY2* show a decline. These findings offer valuable insights into the pathogenesis of Chinese hickory dry rot disease and potential strategies for improving resistance.

## 1. Introduction

*Botryosphaeria dothidea* is a fungus classified in the phylum Ascomycota, class Dothideomycetes, order Botryosphaeriales, family Botryosphaeriaceae, and genus *Botryosphaeria* [1,2]. It is a weak parasite with strong saprophytic capabilities and is considered a dominant pathogen [3,4]. The fungus has a prolonged latent period during infection and typically invades when the host plant is weakened or injured. This can lead to a range of symptoms, including cankers, resin exudation, branch wilting, and fruit rot, all of which significantly affect agricultural productivity and economic development [5].

Chinese hickory (*Carya cathayensis*) is a deciduous tree in the genus *Carya* of the family Juglandaceae. It is primarily found in regions such as Ningguo in Anhui Province and Lin’an, Chun’an, and Tonglu in Zhejiang Province. As a unique oil and nut tree species native to China, Chinese hickory has become a key component of the local economy in northwest Zhejiang and southern Anhui. The development of this industry has not only boosted the income of local residents but has also contributed to the agricultural economy of western Zhejiang and southern Anhui [4]. Like pears, peaches, and apples [6,7,8,9], Chinese hickory is highly susceptible to infection by *B*. *dothidea*. In recent years, however, unscientific fertilization practices aimed at achieving higher yields have led to significant soil degradation in Chinese hickory orchards [10]. This has resulted in soil acidification [11], reduced soil fertility, and a decline in microbial diversity [12], all of which weaken the tree’s resistance to diseases. Consequently, *B. dothidea* has been able to exploit these vulnerabilities, leading to the widespread occurrence of dry rot disease in Chinese hickory. This has caused considerable yield losses and economic setbacks for local farmers, impacting agricultural production in the region. Currently, the main methods for controlling dry rot disease include scraping off infected tissue, applying chemical treatments, developing microbial formulations for biological control using dominant pathogenic strains, and other similar strategies. However, these approaches only manage or alleviate the disease rather than fully eradicate it. Therefore, a deeper understanding of the infection mechanisms of *B. dothidea* in Chinese hickory is essential for developing more effective, targeted strategies for disease prevention and ensuring the sustainable development of this important industry.

During the interaction between plants and pathogens, the pathogen releases a variety of effector molecules to infect the host plant. In response, the plant initiates specific defense mechanisms to prevent pathogen invasion and protect itself from disease. The plant immune system is primarily composed of two types of immune responses. The first is the pattern recognition receptor (PRR), which detects pathogen-associated molecular patterns (PAMPs). This triggers a response known as PAMP-triggered immunity (PTI), which serves as the plant’s first line of defense. The PTI response is relatively simple and involves mechanisms such as calcium ion (Ca^2+^) signaling, hormone regulation, and antioxidant responses, among others [13,14]. This defense system provides plants with the ability to fend off most pathogens. However, some pathogens have evolved mechanisms to inhibit PTI, including the secretion of specific effector molecules. These effectors are delivered to specific cellular locations, where they interfere with the immune responses triggered by PAMPs, leading to a phenomenon known as effector-triggered susceptibility (ETS). At this stage, the plant immune system activates a second line of defense known as effector-triggered immunity (ETI). ETI relies on effector-recognition receptors to detect the pathogen’s effector molecules. Compared to PTI, ETI is faster and more specific. Once an effector is recognized, the plant cell rapidly initiates a series of immune responses, including the activation of protein kinases, programmed cell death, the synthesis and release of plant hormones, and other defense mechanisms. These responses limit the spread and reproduction of pathogenic microorganisms. Additionally, the synthesis of plant metabolites and their associated metabolic pathways play an important role in defending against pathogen invasion. Among these, the phenylpropanoid metabolism pathway is particularly significant, as it produces a range of bioactive compounds that help the plant resist infections [15,16]. One of the key products of this pathway is the plant hormone salicylic acid, which is essential for enhancing both local and systemic resistance to pathogens [17,18].

Currently, research related to plant–pathogen interactions still focuses on the role of protein-coding genes in biological stress response signaling pathways. This includes our laboratory’s previous work on the infection of indoor-grown Chinese hickory trees by *B. dothidea* [19], which only involved mRNA. However, recent advances in high-throughput gene sequencing and large-scale transcriptomic analysis have shown that a significant portion of eukaryotic genomes is transcribed into non-protein-coding RNAs, known as non-coding RNAs (ncRNAs). These ncRNAs respond to different environmental stimuli and participate in the regulation of gene expression to cope with both abiotic and biotic stresses [20,21,22,23]. Studies have found traces of non-coding RNAs, such as small RNAs and long non-coding RNAs, in plant immune systems [24,25]. These RNA molecules can regulate plant immune responses in various ways, such as promoting or inhibiting gene transcription and modulating plant hormone synthesis and signaling pathways [26,27]. Furthermore, previous studies on the interaction between Chinese hickory and *B. dothidea* in controlled indoor environments, although advantageous in controlling experimental variables and reducing external interference, do not fully replicate the natural conditions under which Chinese hickory resist *B. dothidea* infection. Outdoor studies, however, can more realistically simulate the entire process of Chinese hickory defending against *B. dothidea* infection and are closer to actual agricultural production conditions. This makes their findings more valuable for practical application and widespread adoption. Therefore, outdoor research plays an irreplaceable role in developing and optimizing disease management strategies for Chinese hickory. It can provide more practical and actionable recommendations for combating pathogen infection, offering greater guidance for agricultural production.

This study employs transcriptomic techniques, including mRNA and non-coding RNA analyses, to comprehensively investigate the dynamic progression of dry rot disease under natural conditions and its primary defense mechanisms against *B*. *dothidea* infection. The findings offer new insights and a solid research foundation for identifying Chinese hickory varieties with enhanced disease resistance and for developing scientifically informed strategies to prevent and control dry rot disease, thereby providing valuable reference significance.

## 2. Results

### 2.1. The Disease Progression of Dry Rot Disease in Outdoor Chinese Hickory

**Figure 1:**
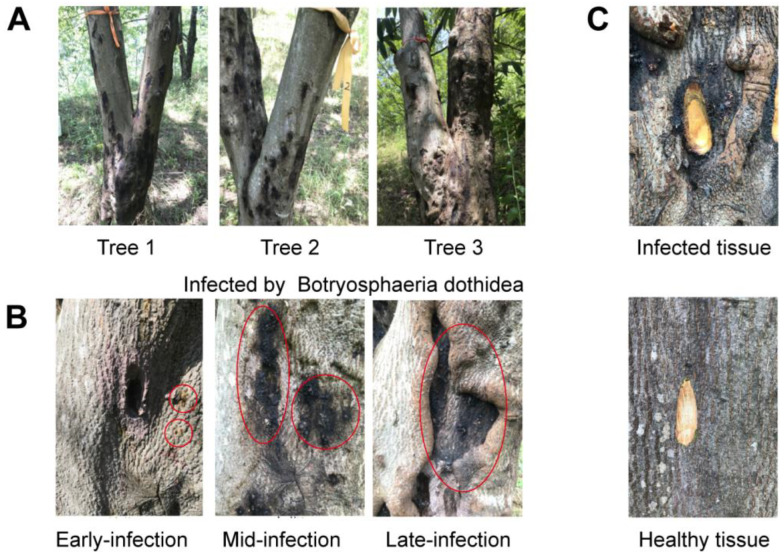
Illustrates the full progression of dry rot disease in outdoor Chinese hickory. (**A**) Three Carya trees with similar age, growth, and health status, labeled Tree 1, Tree 2, and Tree 3 from left to right, were selected for this study. (**B**) The progression of disease in the same area of the tree trunk at different stages of infection: early-infection (24 March 2022), mid-infection (26 May 2022), and late-infection (5 August 2022) stages. The red circle marks the lesion area on the Chinese hickory seedlings as *B. dothidea* infection progresses over time; (**C**) Sampling locations of infected and healthy tissues.

During the early infection stage, yellow-brown spots appeared in the diseased area, with no other obvious symptoms. In the mid-infection stage, numerous canker-like black spots were observed on the tree trunks, and the affected area showed noticeable enlargement. By the late infection stage, the diseased area had developed large, black depressions, with healing tissue visible at the lesion edges (Figure 1B). In contrast, the healthy bark of the control trees remained gray-brown, with a smooth and natural surface throughout all three stages (Figure 1C).

### 2.2. Transcriptomics Profiling of Chinese Hickory with Dry Rot Disease

The transcriptomes of infected tissues from different stages of Chinese hickory were sequenced and categorized into three groups: early infection (Cc_P_D), mid infection (Cc_M_D), and late infection (Cc_A_D). Healthy tissues from each stage were used as control groups (Cc_P_W, Cc_M_W, Cc_A_W). Three biological replicates were included for each treatment group. In total, 18 cDNA libraries were sequenced, generating 234.57 GB of raw data. After quality evaluation and filtering, 5.86 GB of data were removed, resulting in 228.71 GB of clean data. Sample Cc_P_D_1 was excluded due to a low mapping rate (0.06%), likely caused by environmental contamination and excessive ulceration in the infected tissue. The overall sequencing error rate was 0.028%, with an average Q30 value of 93.75%, indicating high-quality data suitable for further analysis (Table S1).

When compared with the reference genome of Chinese hickory, about 75% of the clean data were successfully aligned, with 95.5% of the reads aligning to unique positions of the reference genome (Table S2). Alignment results indicated that 61.95% of the reads were located in exonic regions, 14.92% in intronic regions, and 23.13% in intergenic regions (Figure S1). Functional annotation of the Chinese hickory genome was performed using the EggNOG-mapper database, providing essential annotation data for subsequent analysis (Table S3).

### 2.3. Differential Analyses Between Infected and Healthy Tissues of Chinese Hickory

To investigate gene expression differences between infected and healthy tissues in Chinese hickory, transcript quantification was performed following alignment, assembly, and transcript filtering. The expression matrix was subsequently normalized, and DEGs were identified using edgeR, with a significance threshold of *p*-value ≤ 0.05 and |log2FoldChange| ≥ 1.

The results revealed that in the early stage of infection, 7754 DEGs were identified between healthy and infected tissues, including 4442 up-regulated and 3312 down-regulated genes. In the middle stage, 5805 DEGs were detected (3320 up-regulated and 2485 down-regulated), while in the late stage, only 1503 DEGs were found (1164 up-regulated and 339 down-regulated). The number of DEGs progressively declined as the disease advanced, with the lowest count observed in the late stage.

**Figure 2:**
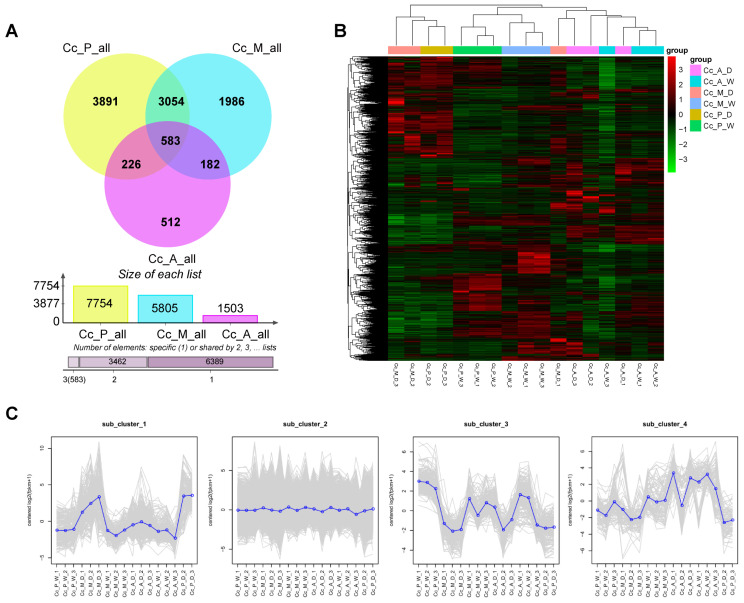
Gene clustering and expression trend analysis of DEGs in Chinese hickory. (**A**) Venn diagram showing DEGs across different comparison groups. Cc_P_all represents the comparison between healthy tissue and infected tissue in the early-infection, Cc_M_all in the mid-infection, and Cc_A_all in the late-infection. This diagram illustrates the number of DEGs unique to each comparison group as well as the number of shared DEGs. The yellow, blue, and purple bar charts display the total number of DEGs for the early, middle, and late infection, respectively, comparing healthy tissue to infected tissue. The stacked bar chart, from left to right, shows the number of DEGs shared by all three groups, shared by two groups, and unique to each individual group. (**B**) Clustering results of DEGs, where each row represents a DEG and each column represents a sample. (**C**) Line plot depicting DEG clustering. A trend analysis of DEG expression patterns across different samples was performed, resulting in the classification of DEGs into four clusters: sub_cluster_1, sub_cluster_2, sub_cluster_3, and sub_cluster_4.

Venn diagram analysis further highlighted stage-specific DEGs, with 3891 unique to the early stage, 1986 to the middle stage, and 512 to the late stage. The number of uniquely up-regulated DEGs was 2105, 960, and 512, respectively, while uniquely down-regulated DEGs were 1940, 1190, and 77, all following a decreasing trend. Additionally, 3637 DEGs were shared between the early and middle stages, 809 DEGs were shared between the early and late stages, and 765 DEGs were shared between the middle and late stages. The greatest overlap of DEGs was observed between the early and middle stages (Figure 2A, Figures S2 and S3).

These findings suggest that in the late stage of infection, the pathogen imposes strong suppression on the plant, leading to the down-regulation of disease-resistance pathways that were actively expressed in the early and middle stages. This suppression likely accounts for the reduced number of DEGs and the increased similarity in gene expression profiles between infected and healthy tissues.

### 2.4. Gene Clustering and Expression Trend Analysis of DEGs in the Infected Chinese Hickory

To investigate the major functions of differentially expressed genes (DEGs) between infected and healthy tissues and their roles in the development of dry rot disease in Chinese hickory (outdoor Chinese hickory), clustering analysis was performed on DEGs from all comparison groups. The results revealed that the samples predominantly grouped into two main clusters (Figure 2B). The first cluster included the early-stage samples (Cc_P_D_2, Cc_P_D_3) and middle-stage samples (Cc_M_D_2, Cc_M_D_3), while the second cluster mainly comprised healthy tissue samples (Cc_P_W, Cc_M_W, Cc_A_W) and late-stage infected tissue samples (Cc_A_D) (Figure 2B). Gene expression patterns in the early and middle-stage disease lesions were similar, whereas the late-stage infected tissues exhibited expression profiles more akin to healthy tissues. These findings were consistent with the DEG count statistics. Additionally, we observed that the second cluster was further divided into two subclusters. The healthy tissue samples from the early and middle stages of infection, Cc_P_W and Cc_M_W, formed one group, while the healthy tissue sample from the late stage, Cc_A_W, and the infected tissue sample from the late stage, Cc_A_D, were grouped together (Figure 2B). This differentiation may be attributed to the severe pathogen infection in the late stage, which suppresses the expression of disease resistance-related genes, thereby disrupting the physiological and metabolic activities of healthy tissues. As a result, the gene expression pattern in these healthy tissues differs from that in the early and middle stages and becomes more similar to that of the infected tissues in the late stage, leading to the formation of a distinct subcluster.

Based on the DEG clustering results, the DEGs were divided into four clusters. Trend analysis of the DEG expression patterns across different samples was performed, and clustering line charts were generated. Cluster 2 contained the largest number of genes (14,743), but their expression patterns did not show significant differences between infected and healthy tissues. Clusters 3 and 4 contained 246 and 100 genes, respectively, with most genes in these clusters exhibiting lower expression levels in infected tissues compared to healthy tissues. Cluster 1, which included 237 DEGs, showed higher expression levels in infected tissues, particularly in the early and middle stages of disease lesions, compared to the late-stage lesions (Figure 2C).

Under normal conditions, certain genes are rapidly activated shortly after pathogen infection, triggering plant defense responses and enhancing disease resistance. To further understand the biological functions and pathways of DEGs in Cluster 1, GO and KEGG enrichment analyses were conducted. The GO enrichment analysis revealed that significant terms were primarily associated with chitin, cell wall synthesis, and related processes (Table S4). A total of 36 DEGs were annotated, including four involved in phenylpropanoid biosynthesis, four in plant hormone signal transduction, three in the MAPK signaling pathway–plant interaction, one in stilbenoid, diarylheptanoid, and gingerol biosynthesis, and one in flavonoid biosynthesis (Table S5). Among the three genes identified in the MAPK signaling pathway plant, *CCA0912S0031* encodes the ethylene receptor protein ETR/ERS, while *CCA0986S0058* and *CCA0108S0011* are associated with basic endochitinase B (Chi B), an enzyme that plays a key role in plant defense by producing chitinase to combat fungal pathogens. The gene *CCA0691S0117* was enriched in the phenylpropanoid, flavonoid, and stilbenoid biosynthesis pathways. It encodes cinnamate-4-hydroxylase (C4H), a crucial enzyme that catalyzes the conversion of cinnamic acid into p-coumaric acid, a key step in these pathways. Additionally, *CCA0533S0074*, annotated in the plant hormone signal transduction pathway, encodes a TGA transcription factor. These enrichment results suggest that during the defense response to *B. dothidea* infection, Chinese hickory enhances lignin synthesis to fortify the cell wall, increases chitinase activity to degrade the pathogen’s cell wall, boosts oxidoreductase activity, and promotes the activity of TGA transcription factors. These mechanisms collectively strengthen the expression of disease resistance-related proteins.

### 2.5. Enrichment Analysis and Identification of Key Genes in Defense Pathways of Chinese Hickory Against Pathogens Across Various Infection Stages

Building on the previous analysis, the gene expression patterns in infected tissues from the early and middle stages of disease development show significant differences compared to healthy tissues. This suggests that these stages may harbor candidate genes involved in resistance to pathogen invasion in Chinese hickory. Since disease-resistant genes may also be present in tissues affected during the late stage of infection, GO functional enrichment and KEGG pathway enrichment analyses were conducted on three comparison groups: Cc_P_D vs. Cc_P_W (early infection), Cc_M_D vs. Cc_M_W (mid infection), and Cc_A_D vs. Cc_A_W (late infection). These analyses aim to identify key pathways involved in regulating pathogen resistance in Chinese hickory, offering insights into the specific roles of genes associated with disease defense throughout the infection process.

**Figure 3:**
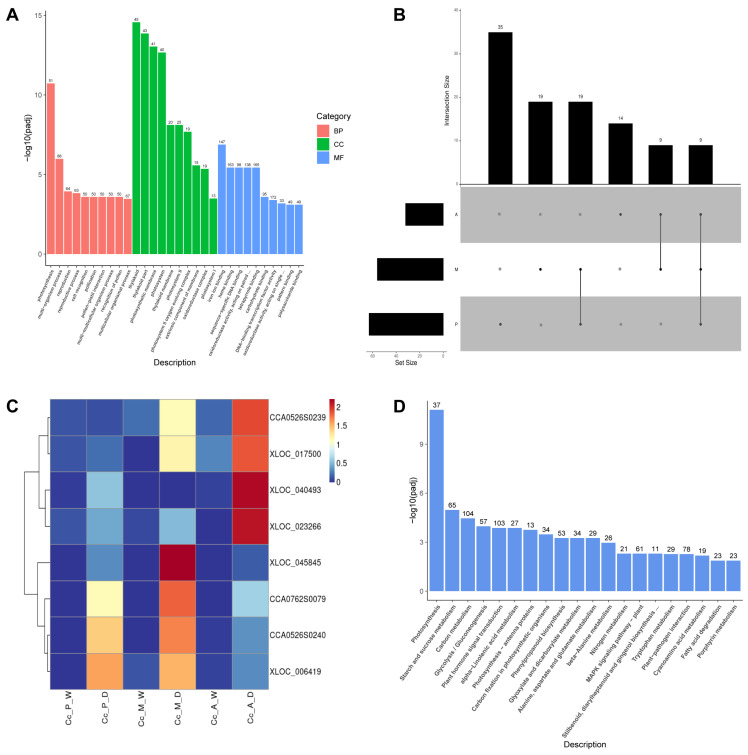
Enrichment analysis of DEGs between comparative groups at different stages. (**A**) GO enrichment analysis in Cc_P_D vs. Cc_P_W. The GO terms significantly enriched in the diseased early-stage infected tissues compared to healthy tissues were analyzed. BP, CC, and MF refer to the biological process, cellular component, and molecular function GO terms, respectively, that were significantly enriched; (**B**) UpSet diagram showing the significantly enriched GO terms between different comparison groups. A, M, and P refer to the late-infection, mid-infection, and early-infection stages. (**C**) Heatmap of disease resistance-related genes. Each row corresponds to a DEG, while each column represents a sample. (**D**) KEGG enrichment analysis in Cc_P_D vs. Cc_P_W. The KEGG pathways significantly enriched between diseased early-stage infected tissues and healthy tissues were compared.

Comparison of the GO enrichment results across the three groups revealed that, aside from the GO terms shared by all three stages, 19 terms were common between the early and middle stages, none were shared between the early and late stages, and 9 terms were shared between the middle and late stages (Figure 3B). A comparison of the GO enrichment results across the three infection stages revealed that 19 GO terms were shared between the early and mid-infection stages, while no terms were common between the early and late-infection stages. In contrast, 9 GO terms were shared between the mid-infection and late-infection stages (Figure 3B). Despite these differences, the GO terms identified across the three stages showed notable similarities, with strong enrichment in oxidoreductase activity, peroxidase activity, and chitin-related enzymes (Figure 3A, Figures S4 and S5). These findings align with the enrichment results for genes in cluster 1, suggesting that Chinese hickory may consistently utilize mechanisms such as enhancing oxidoreductase activity, reinforcing the cell wall, and degrading fungal cell walls to combat pathogen infection throughout disease progression.

The biological process terms enriched in the early and mid-infection stages were distinct from those in the late-infection stage. In the early and mid stages, terms were primarily associated with photosynthesis and other general biological processes, whereas the late-stage terms were predominantly focused on plant defense responses to biotic stress. Interestingly, defense-related GO terms were present during the early and mid-infection stages as well, with a comparable number of enriched genes to those found in the late-infection stage. The lack of significant enrichment in the early stages is likely due to differences in the size of the foreground differential gene set used for GO enrichment analysis. Overall, examining the genes linked to defense against external stimuli across all three stages provides valuable insights into the defense strategies employed by Chinese hickory to resist pathogen infection.

The analysis of genes associated with defense-related terms in response to external stimuli identified the presence of chitin recognition protein genes and the *NPR1* gene (non-expressor of pathogenesis-related gene 1). After deduplication and normalization of these genes, an expression heatmap was generated (Figure 3C). The results revealed significant differences in expression patterns among the eight genes across the various infection stages. For example, NPR1 (*CCA0526S0239*) and *XLOC_017500* were predominantly expressed during the middle and late infection stages, whereas genes such as the chitin recognition protein gene (*CCA0762S0079*) and another NPR1 (*CCA0526S0240*) were more highly expressed in the early and middle stages. These findings suggest that the metabolic pathways and signaling mechanisms involved in Chinese hickory’s resistance to pathogen infection may differ depending on the stage of disease progression.

The chitin recognition protein gene, in particular, shows high expression during the early and middle infection stages, which likely facilitates the rapid detection of pathogen-associated molecular patterns (PAMPs). This heightened expression is thought to trigger immune responses that help the plant quickly suppress pathogen spread during the initial phase of infection.

To further investigate the key biochemical metabolic pathways and signal transduction pathways associated with differentially expressed genes (DEGs) at various stages of Chinese hickory dry rot disease, we utilized KEGG pathway annotation to classify the DEGs. A padj value of less than 0.05 was considered indicative of significant enrichment. The KEGG enrichment analysis revealed that genes associated with the photosynthesis pathway exhibited the most significant differential expression in the early infection stage comparisons. Additionally, the carbon metabolism and plant hormone signal transduction pathways contained the largest number of DEGs.

In the middle and late infection stages, the phenylpropanoid biosynthesis pathway showed the most significant DEG enrichment, while the plant hormone signal transduction pathway contained the greatest number of DEGs (Figure 3D, Figures S6 and S7). The significantly enriched pathways unique to the three infection stages were 18, 3, and 1, respectively. Commonly enriched pathways across all stages included four key pathways: photosynthesis, carbon fixation in photosynthetic organisms, phenylpropanoid biosynthesis, and nitrogen metabolism.

During the early and middle infection stages, 15 significantly enriched pathways were shared, including several plant defense-related pathways such as plant hormone signal transduction, phenylpropanoid biosynthesis, MAPK signaling pathway–plant, plant–pathogen interaction, flavonoid biosynthesis, stilbenoid/diphenylheptane and gingerol biosynthesis, and alpha-linolenic acid metabolism (Figure S8 and Table S6). This suggests that the early and middle infection stages are critical periods for the activation of plant defense pathways. In contrast, metabolic pathways related to plant defense substances, plant–pathogen interactions, and signaling pathways showed limited or no significant enrichment during the late infection stage. This indicates a weakened resistance to pathogen invasion during the late infection period.

**Figure 4:**
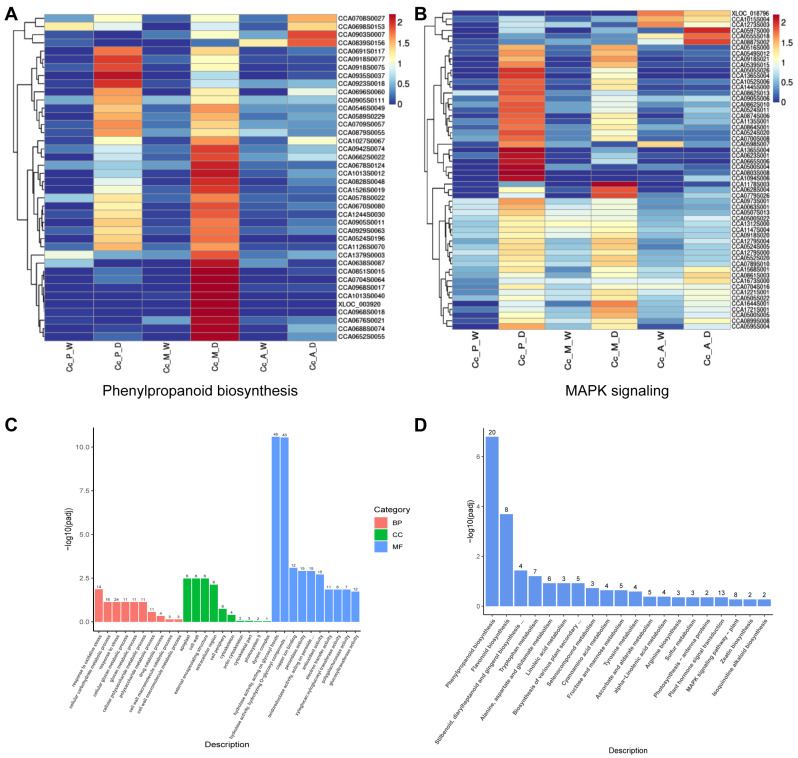
Identification of the key genes involved in Chinese hickory’s defense pathways against pathogens. (**A**,**B**) Gene expression profiles of disease-resistant pathways: (**A**) Phenylpropanoid biosynthesis; (**B**) MAPK signaling. Each row corresponds to a DEG, while each column represents a sample; (**C**) GO enrichment analysis of up-regulated genes in Cc_M_D vs. Cc_P_D. The GO enrichment terms for up-regulated genes in diseased middle-stage infected tissues compared to diseased early-stage infected tissues were analyzed. BP, CC, and MF refer to the biological process, cellular component, and molecular function GO terms, respectively, that were significantly enriched; (**D**) KEGG enrichment analysis of up-regulated genes in Cc_M_D vs. Cc_P_D. The KEGG enrichment pathways of up-regulated genes in diseased middle-stage infected tissues were compared with those in diseased early-stage infected tissues.

The gene expression levels of pathways involved in phenylpropanoid biosynthesis, MAPK signaling pathway–plant, plant–pathogen interaction, plant hormone signal transduction, flavonoid biosynthesis, as well as stilbenoid/diphenylpropane/diarylpropane biosynthesis, and gingerol biosynthesis, were normalized to construct gene expression profiles (Figure 4A,B and Figures S9–S12).

Based on the gene expression profiles, flavonoid biosynthesis was found to be the pathway with the most consistent expression across the three time points. In contrast, pathways such as phenylpropanoid biosynthesis, MAPK signaling pathway–plant, plant–pathogen interaction, stilbenoid, diphenylpropane/diphenylheptane biosynthesis, gingerol biosynthesis, and plant hormone signaling exhibited significantly higher expression during the early and middle disease stages compared to the late stage. This suggests that the defense response of Chinese hickory against the pathogen varies distinctly at different stages of disease progression.

To further explore the regulatory responses of Chinese hickory during disease progression, this study introduces two additional comparison groups: infected tissue in the middle stage vs. the early stage (Cc_M_D vs. Cc_P_D) and infected tissue in the late stage vs. the middle stage (Cc_A_D vs. Cc_M_D). By comparing the differentially expressed genes (DEGs) in infected tissues across these infection stages, we aim to identify key phases in the defense response of Chinese hickory and examine how defense pathways and disease-resistance genes vary. GO and KEGG enrichment analyses were conducted separately for up-regulated and down-regulated genes in these comparison groups (Cc_M_D vs. Cc_P_D and Cc_A_D vs. Cc_M_D) (Figure 4C,D and Figures S13–S18). The peak of dry rot disease occurs between April and May when *B. dothidea* has caused significant wood damage and black lesions on the bark [28]. In the mid-infection period (May), compared to the early-infection period (March), up-regulated genes associated with lignin biosynthesis, cell wall strengthening, and peroxidase activity were observed, contributing to reinforcement of the cell wall and defense against pathogen invasion. Additionally, genes linked to oxidoreductase activity and copper ion binding were up-regulated, enhancing reactive oxygen species (ROS) production and boosting Chi B activity, which strengthens the defense against fungal infection. Additionally, the expression of genes involved in phenylpropanoid biosynthesis and related pathways increased, possibly as a response by Chinese hickory to enhance the production of disease-resistant compounds. However, an analysis of enriched pathways down-regulated in the late stage compared to the middle stage revealed that most disease resistance-related pathways enriched in the early and middle stages were significantly down-regulated in the late stage. These include key pathways such as phenylpropanoid biosynthesis and plant hormone signal transduction.

### 2.6. Building Dry-Rot Disease Resistance Pathways in Chinese Hickory

To further investigate the primary products and genes involved in the defense response of outdoor Chinese hickory against *B. dothidea* invasion, this study summarizes key resistance-related pathways, highlighting significant genes and their associated metabolites.

**Figure 5:**
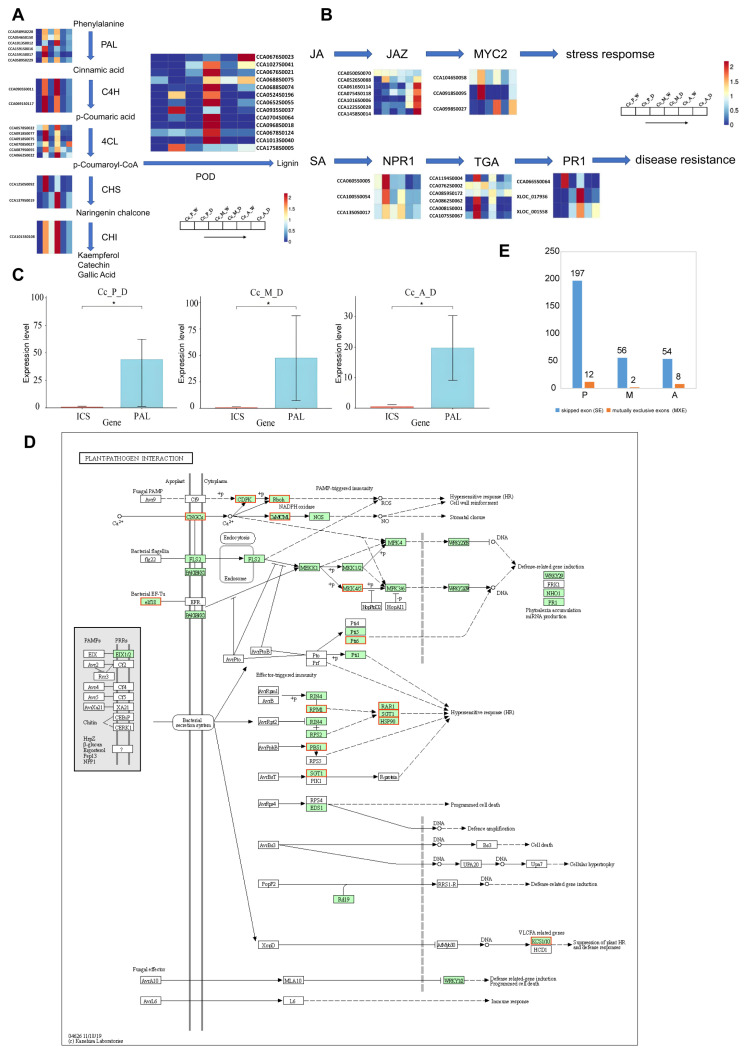
Disease-resistance-related pathways and differential alternative splicing. (**A**) Diagram of the phenylpropanoid biosynthesis pathway. This figure illustrates the key pathways involved in phenylpropanoid biosynthesis and highlights the expression changes in genes associated with these pathways during disease progression in Chinese hickory. (**B**) Expression of genes related to the SA and JA pathways. This figure presents the expression patterns of genes related to the SA and JA signaling pathways during the disease progression in *Carya cathayensis*. In the heatmaps of Figures (**A**,**B**), each row represents an individual gene, and each column corresponds to an experimental group. The groups, from left to right, are as follows: healthy tissue at the early stage of infection, infected tissue at the early stage of infection, healthy tissue at the middle stage of infection, infected tissue at the middle stage of infection, healthy tissue at the late stage of infection, and infected tissue at the late stage of infection. (**C**) Differential expression analysis of PAL-related and ICS-related genes at different stages. * denotes a significant difference between the two groups. (**D**) KEGG pathway map of plant–pathogen interactions, with red borders highlighting up-regulated DEGs. (**E**) Statistics of significant alternative splicing events. Statistics of significant alternative splicing events are presented, highlighting those identified at different disease stages. P, M, and A correspond to the early, middle, and late stages of infection, respectively. Blue indicates skipped exons, while orange denotes mutually exclusive exons.

The phenylpropanoid biosynthesis pathway produces essential compounds such as lignin and flavonoids, which play critical roles in plant growth, development, and stress resistance [29]. Core enzymes in this pathway, including phenylalanine ammonia-lyase (PAL), 4-coumarate-CoA ligase (4CL), and C4H, regulate the initial reactions and play a decisive role in pathway expression [30]. These enzymes also control two important branches of the phenylpropanoid pathway: flavonoid biosynthesis and the production of stilbenoids, diarylheptanoids, and gingerol. To provide a clearer representation of these key genes, this study simplified the biosynthetic pathways, integrating the essential genes and metabolites involved (Figure 5A).

Across the three infection stages, most genes exhibited significantly higher expression levels in infected tissues compared to normal tissues. Furthermore, expression levels in infected tissues were higher during the early and middle stages of infection than in the late stage. The expression levels of the three key enzyme-related genes remained relatively consistent in infected tissues during the early and middle stages. However, peroxidase-related genes showed notably higher expression levels in infected tissues during the middle stage compared to the early stage. This suggests that, during the middle stage of infection, Chinese hickory may accelerate lignin synthesis to counteract the severe tissue damage caused by *B. dothidea*.

Salicylic acid (SA) is a crucial plant hormone that regulates various physiological processes, including disease resistance, growth and development, and responses to stress [31]. In plants, SA is primarily synthesized through two pathways: the phenylalanine ammonia-lyase (PAL) pathway and the isochorismate synthase (ICS) pathway. However, the predominant pathway for SA synthesis can vary among different plant species. To identify the main SA synthesis pathway in Chinese hickory, the relative expression levels of key genes involved in both the PAL and ICS pathways were analyzed. The results revealed that, across the three stages of disease progression, the expression of *PAL* genes in the infected tissue was significantly higher than that of *ICS* genes (Figure 5C). This suggests that, in Chinese hickory, the PAL pathway is likely the primary route for SA synthesis following pathogen infection.

SA and jasmonic acid (JA) play distinct yet complementary roles in plant defense. SA is primarily involved in systemic acquired resistance, which targets biotrophic pathogens that rely on living host tissues for survival. In contrast, JA is mainly associated with local defense responses against necrotrophic pathogens that feed on dead plant tissue [32]. JAZ (jasmonate zim domain) proteins act as inhibitors in the JA signaling pathway by preventing JA from binding to the transcription factor COI1, thereby blocking the transmission of the jasmonic acid signal.

Analysis of the heatmap depicting JAZ-related gene expression shows that these genes are not highly expressed in the disease spots during the early and middle stages of infection. However, their expression significantly increases in the later stages of infection. Simultaneously, MYC2, a transcription factor that promotes the JA pathway, exhibits weak expression at the late stage. This suggests that the JA pathway is being suppressed during the later stages of infection (Figure 5B).

In the SA signaling pathway, a pathogen attack triggers the activation of the NPR1 protein, which then translocates from the cytoplasm to the nucleus. In the nucleus, NPR1 interacts with TGA transcription factors to form the NPR1-TGA complex, which initiates the plant’s immune response. TGA transcription factors enhance disease resistance by binding to the promoter of the SA-induced gene *PR1* (pathogenesis-related protein), thus promoting *PR1* gene expression [33,34,35]. In this study, the expression of genes related to NPR1, TGA, and PR1 was significantly higher in the disease spot tissues during the early and middle stages of infection compared to the late stage. This suggests that, in the later stages of infection, the SA pathway and its associated disease-resistance genes are suppressed (Figure 5B).

In the KEGG analysis of up-regulated genes in the comparison between Cc_A_D and Cc_M_D, the plant–pathogen interaction pathway was significantly enriched. However, within the overall gene expression profile of this pathway, most genes showed higher expression during the early and mid stages of infection compared to the late stage (Figure 5C). To investigate the changes in relevant genes during the late infection stage, a signaling pathway diagram was created to highlight the specific locations and functions of the up-regulated genes within the pathways (Figure 5D). The results indicate that during the late infection stage, up-regulated genes are predominantly involved in the Ca^2+^ signaling pathway, including calcium-dependent protein kinase (CDPK), respiratory burst oxidase (Rboh), and calmodulin (CAM). These genes play key roles in promoting the production of reactive oxygen species (ROS), which, in turn, trigger hypersensitive responses (HR) in plants. The up-regulation of these genes may reflect Chinese hickory’s continued attempt to resist pathogens in the late stage of infection by enhancing ROS production, underscoring the complexity of its disease resistance mechanism.

### 2.7. Differential Alternative Splicing

In the process of plant disease resistance, alternative splicing plays a crucial role in regulating gene function and expression by modulating transcriptional processing to produce different splice isoforms. This, in turn, affects the plant’s response to and resistance against pathogens. In this study, we used rMATS (4.0.2) software to analyze alternative splicing events in Chinese hickory during disease-susceptible periods. The analysis focused on several types of splicing events, including skipped exons (SE), retained introns (RI), mutually exclusive exons (MXE), alternative 5′ splice sites (A5SS), and alternative 3′ splice sites (A3SS).

By examining alternative splicing events across three disease-susceptible stages, a total of 11,179, 7750, and 8480 splicing events were identified in the early, middle, and late disease stages, respectively. These events were primarily characterized by exon skipping and mutually exclusive exons (Figure 5E). After applying a significance filter, 209, 58, and 57 Differential Alternative Splicing (DAS), events were identified in the early, middle, and late stages, respectively, originating from 199, 57, and 53 genes. The number of alternative splicing events in the early disease stage was significantly higher than in the middle and late stages.

Additionally, the early disease stage also exhibited the highest number of differentially expressed genes (DEGs), while the late stage had the lowest number of DEGs. This suggests that, during the early disease stage, Chinese hickory experiences environmental stress and responds by producing a large amount of RNA, including through alternative splicing, to regulate gene expression.

KEGG enrichment analysis of the genes involved in alternative splicing revealed that 64 genes were annotated to relevant pathways, including disease-related pathways such as phenylalanine metabolism, biosynthesis of phenylalanine, tyrosine, and tryptophan, isoquinoline alkaloid biosynthesis, ascorbate and aldarate metabolism, peroxisome pathways, and the MAPK signaling pathway in plants. A summary of the genes involved in these pathways (Table S7) indicated that the early disease stage had the highest number of alternative splicing events, the majority of which were positively regulated. This suggests that, during early pathogen invasion, the plant responds rapidly by initiating a series of defense reactions, including post-transcriptional regulation and alternative splicing.

### 2.8. Investigation of the Regulatory Mechanisms of lncRNA in Chinese Hickory Dry Rot Disease

**Figure 6:**
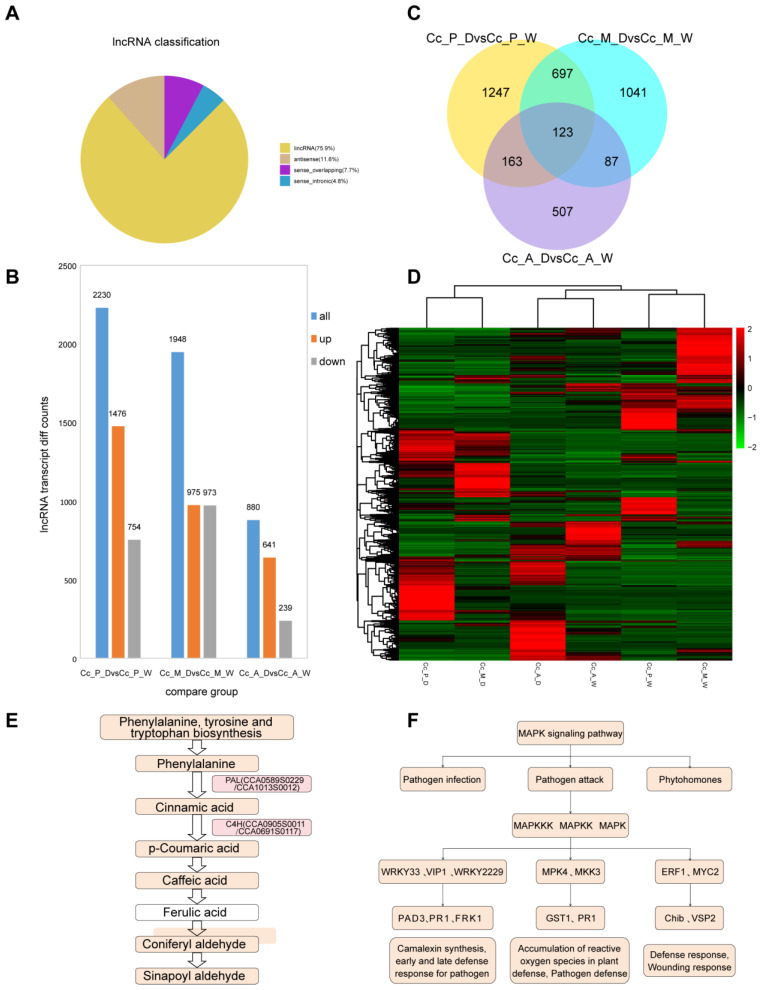
Regulatory analysis of lncRNA. (**A**) Distribution of lncRNA types. Yellow, brown, purple, and blue represent the proportions of lincRNA, antisense, sense-overlapping, and sense-intronic, respectively. (**B**) Number of DEGs (Differentially Expressed Genes) among different comparison groups for lncRNA. The gray bars represent the total number of DEGs, the blue bars indicate up-regulated DEGs, and the orange bars show down-regulated DEGs. (**C**) Venn diagram showing DEGs of lncRNA across different comparison groups. (**D**) Clustering results of lncRNA DEGs. Each row corresponds to a DEG, while each column represents a sample. (**E**,**F**) lncRNA disease resistance genes involved in Chinese hickory dry rot disease interaction. (**E**) Phenylpropanoid biosynthesis pathway. (**F**) Plant MAPK signaling metabolic pathway.

Clean reads were assembled and merged using StringTie (version 1.3.3b), and long non-coding RNAs (lncRNAs) were identified using gffcompare (version 0.10.6) software, with further analysis through CPC, CNCI, and FPKM metrics. A total of 23,699 lncRNAs were detected, with lincRNAs comprising the majority at approximately 76%. Antisense, sense overlapping, and sense intronic lncRNAs accounted for smaller proportions, each making up less than 12% of the total (Figure 6A). Differentially expressed lncRNAs (DEGs) were identified between different comparison groups using the edgeR screening criteria (*p*-value ≤ 0.05, |log2FoldChange| ≥ 0.0). The results showed that in the Cc_P_D vs. Cc_P_W comparison, 2230 DEGs were identified (1476 up-regulated, 754 down-regulated); in Cc_M_D vs. Cc_M_W, 1948 DEGs were found (975 up-regulated, 973 down-regulated); and in Cc_A_D vs. Cc_A_W, 880 DEGs were observed (641 up-regulated, 239 down-regulated) (Figure 6B).

The unique DEGs for the early, middle, and late disease stages were 1247, 1041, and 507, respectively, with a total of 123 common DEGs across all stages. The highest number of shared DEGs was found between the early and middle disease stages, with 697 DEGs common to both (Figure 6C). This pattern parallels the DEG results for mRNA comparison groups. As the disease progresses in Chinese hickory, the number of DEGs between infected and healthy tissues decreases, suggesting that the gene expression profiles for pathogen resistance in the early and middle stages are more similar. In the subsequent lncRNA DEG clustering analysis, infected tissues from the early and middle disease stages clustered together, while the infected tissue from the late stage grouped with the normal tissue (Figure 6D).

By analyzing the co-location and co-expression relationships between lncRNAs and protein-coding genes, a total of 270,254 co-expressed lncRNA target genes and 114,152 co-localized lncRNA target genes were identified. To further investigate the functional roles of these target genes, GO and KEGG enrichment analyses were performed. In the GO enrichment analysis for the three comparison groups—Cc_P_D vs. Cc_P_W, Cc_M_D vs. Cc_M_W, and Cc_A_D vs. Cc_A_W—GO terms related to cell wall organization, oxidoreductase activity, and other relevant categories were enriched (Tables S8–S13). The KEGG pathway analysis revealed functional enrichment patterns similar to those observed for mRNA, with the most significantly enriched pathways in the early and middle disease stages, including plant MAPK signaling and phenylpropanoid biosynthesis (Tables S14–S19). These findings further support that, in response to *B. dothidea* infection, Chinese hickory primarily employs defense strategies such as reinforcing the cell wall and enhancing oxidoreductase activity. Notably, the most critical phases of gene expression related to disease resistance occur during the early and middle stages of the disease.

To further explore the lncRNA-related disease resistance genes involved in the interaction with Chinese hickory wood rot disease, we selected two significantly expressed metabolic pathways, KEGG: jre00940 and KEGG: jre04016, for further investigation. In the biosynthetic pathway of phenylalanine, tyrosine, and tryptophan in the phenylpropanoid biosynthesis pathway, phenylalanine is first converted into cinnamic acid, which then undergoes a series of reactions to synthesize sinapoyl aldehyde. Sinapoyl aldehyde can be converted into lignin monomers and further synthesized into lignin (specifically syringyl lignin). We observed a large number of differentially expressed genes (DEGs) targeting lncRNAs in this pathway when comparing diseased and healthy tissues, including PAL-related genes (*CCA0589S0229*, *CCA1013S0012*), C4H-related genes (*CCA0905S0011*, *CCA0691S0117*), and others (Figure 6E).

In the MAPK signaling metabolic pathway, three key pathways related to pathogen resistance were identified: pathogen infection, pathogen invasion, and plant hormone signaling. We found many DEGs in these pathways as well, including transcription factors like WRKY33 (*CCA0524S0201*, *CCA0700S0083*), VIP1 (*CCA0829S0093*), WRKY2229 (*CCA0628S0043*, *CCA0874S0068*, *CCA0623S0083*, *CCA1461S0015*, *CCA0782S0162*), MKK3 (*CCA0520S0027*), ERF1 (*CCA0912S0015*, *CCA0510S0116*, *CCA0757S0061*, *CCA0888S0004*), and MYC2 (*CCA1046S0058*, *CCA1055S0039*, *CCA0982S0067*, *CCA0846S0036*, *CCA0918S0095*), as well as genes related to plant defense responses such as PR1 (*CCA0665S0064*, *CCA0870S0007*, *CCA1430S0006*, *XLOC_001555*, *XLOC_017937*, *XLOC_017936*, *XLOC_017938*) and ChiB (*CCA0507S0206*, *CCA0986S0058*, *CCA0108S0011*), among others (Figure 6F, Table S23). Additionally, we found that many of these genes overlap with DEGs from the same pathways in mRNA expression.

### 2.9. Regulatory Analysis of circRNA in Chinese Hickory Dry Rot Disease

**Figure 7:**
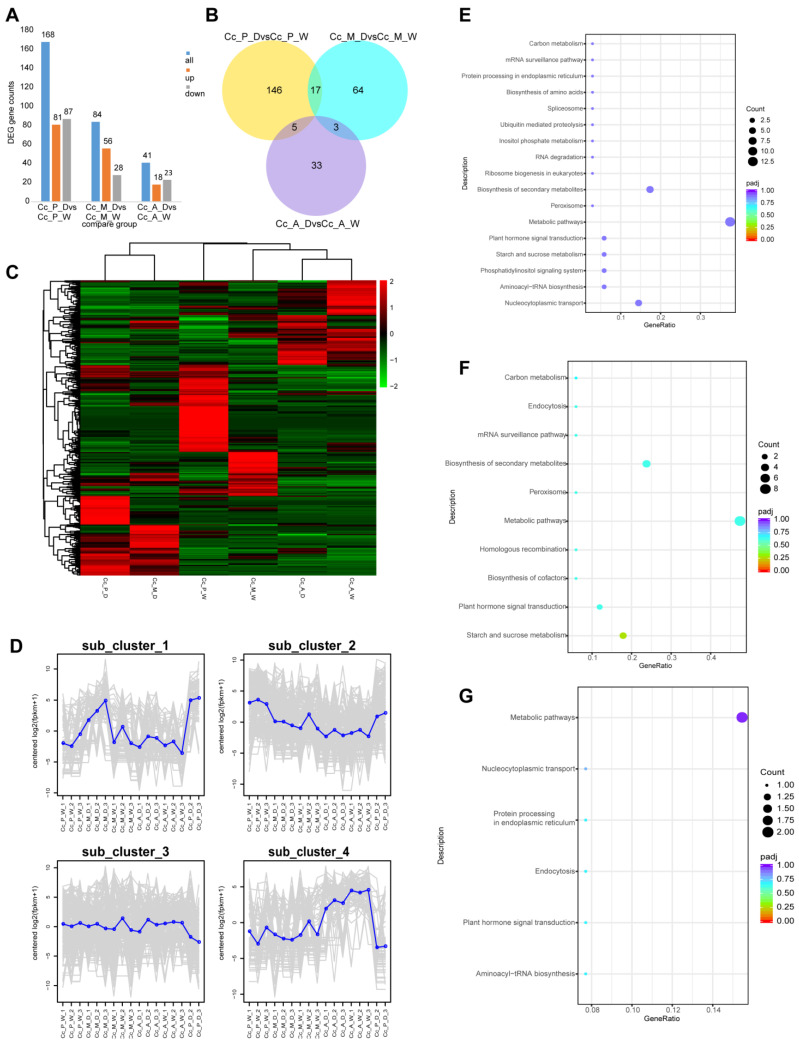
Regulatory analysis of circRNA. (**A**) Number of DEGs in circRNA across different comparison groups. The gray bars represent the total number of DEGs, the blue bars indicate up-regulated DEGs, and the orange bars show down-regulated DEGs; (**B**) Venn diagram showing DEGs of circRNA across different comparison groups; (**C**) Clustering results of circRNA DEGs. Each row corresponds to a DEG, while each column represents a sample; (**D**) Clustering line graph of circRNA DEGs. A trend analysis of DEG expression patterns across different samples was performed, resulting in the classification of DEGs into four clusters: sub_cluster_1, sub_cluster_2, sub_cluster_3, and sub_cluster_4; (**E**–**G**) Significantly enriched pathways between healthy tissue and infected tissue comparison groups during early-infection, mid-infection, and late-infection stages.

By combining CIRI and find_circ analysis, a total of 4253 circRNAs were identified. The expression levels of these circRNAs were assessed based on read counts. Differential expression analysis was then conducted using edgeR, with a *p*-value ≤ 0.05 and |log2FoldChange| ≥ 0.0 as criteria for identifying significant circRNAs. The differentially expressed genes (DEGs) in the control groups at the early, middle, and late disease stages were as follows: 168 DEGs in the early stage (81 up-regulated, 87 down-regulated); 84 DEGs in the middle stage (56 up-regulated, 28 down-regulated); and 41 DEGs in the late stage (18 up-regulated, 23 down-regulated) (Figure 7A). The unique DEGs for each stage were 146 for early, 64 for middle and 33 for late disease stages. Shared DEGs included 17 between the early and middle stages, 5 between the early and late stages, and 3 between the middle and late stages (Figure 7B).

The number of DEGs in circRNAs decreased as the outdoor Chinese hickory dry rot disease progressed. Notably, only the middle disease stage exhibited more up-regulated DEGs than down-regulated, suggesting that the resistance of outdoor Chinese hickory to *B. dothidea* infection initially increases but then declines over time. The DEGs shared between the early and middle stages were also the most abundant in circRNAs.

Clustering analysis of circRNA differential expression between healthy and infected samples at the early, middle, and late disease stages revealed two distinct clusters: one corresponding to early- and middle-stage infected tissues and the other to late-stage infected tissues and healthy tissues (Figure 7C). Based on this clustering, the DEGs were further divided into four sub-clusters. In sub-cluster 1, expression levels in infected tissues were higher than in healthy tissues, with significantly higher expression in the early and middle disease stages compared to the late stage. Conversely, sub-cluster 4 exhibited higher expression in healthy tissues than in infected tissues, with significantly higher expression in the late disease stage. Therefore, sub-cluster 1 and sub-cluster 4 were selected for further analysis (Figure 7D).

We performed KEGG functional enrichment analysis on the differentially expressed circRNAs, using a threshold of *padj* < 0.05. Analysis of the KEGG enrichment results for the comparisons Cc_P_D vs. Cc_P_W, Cc_M_D vs. Cc_M_W, and Cc_A_D vs. Cc_A_W revealed that the late infection group had fewer significantly enriched pathways compared to the early and middle infection groups. However, the three comparison groups shared common pathways related to plant disease resistance, including metabolic pathways and plant hormone signal transduction. Among these, metabolic pathways were the most significantly enriched across all three groups. Furthermore, in the comparisons between the early and middle infection stages, shared pathways related to disease resistance included peroxisome, secondary metabolite biosynthesis, and carbon metabolism (Figure 7E–G). Notably, the disease resistance patterns in circRNAs were more similar between the early and middle infection stages.

To identify circRNAs associated with the interaction between Chinese hickory trees and wood rot disease, we further investigated the significantly enriched pathways. In the plant hormone signaling pathway, which was notably enriched across all disease stages (early, middle, and late), we found differentially expressed genes (DEGs) in the salicylic acid signaling pathway during the early and middle stages of infection, such as NPR1 (*CCA0953S0061*) and TGA (*CCA1075S0067*) (Table S23). However, no DEGs were detected in this pathway during the late stage of infection. We hypothesize that during the initial stages of infection, Chinese hickory trees mount a strong defense response against the pathogen. As the infection progresses and the pathogen invades deeper, many of the genes in the disease resistance pathways are suppressed. This would explain the lack of DEGs in the late infection stage, which is consistent with previous mRNA studies.

The biosynthesis of secondary metabolites was significantly enriched in the early and middle infection stages but not in the late stage. In this pathway, the differentially expressed circRNAs were primarily involved in the synthesis of antibiotic-like secondary metabolites such as ansamycins, polyketides, and monoterpenoids. We speculate that during the early and middle stages of infection, Chinese hickory trees produce secondary metabolites with antibiotic properties to inhibit the pathogen’s cell wall formation and protein synthesis, thus reducing its activity.

## 3. Discussion

The kernel of Chinese hickory is rich in proteins, essential minerals, α-tocopherol (vitamin E), and polyunsaturated fatty acids [36], making it a highly nutritious and economically valuable food. This has garnered significant attention due to its health benefits and growing market demand. However, *B. dothidea*, the pathogen responsible for dry rot disease in Chinese hickory, poses a serious threat to its cultivation and production. Despite the crop’s economic importance, research on controlling Chinese hickory dry rot disease remains limited, and effective management strategies are urgently needed to mitigate its impact on hickory orchards.

Previous studies have shown that dry rot disease in Chinese hickory is primarily caused by the fungus *B. dothidea* [37]. During its infection process, the fungus produces a range of enzymes that degrade the plant cell wall, disrupting cellular structures and utilizing the plant’s nutrients for its own growth and reproduction. Additionally, the pathogen secretes toxins and other virulence factors that impair the plant’s metabolic and physiological functions, ultimately making the plant more susceptible to disease or even causing its death [38,39].

In this study, we selected three outdoor Chinese hickory trees with similar growth and health conditions and collected tissue samples from both diseased and healthy plants at different stages of the dry rot disease (early, middle, and late stages). These samples were subjected to transcriptomic analysis to explore the key pathways involved in Chinese hickory disease resistance.

During the early stages of infection, plants can initiate immune responses mediated by Pattern-Recognition Receptors (PRRs), which recognize pathogen-associated molecular patterns (PAMPs) from the invading pathogen. For example, fungal chitin and ergosterol are detected by plant PAMP receptors, thereby triggering the plant’s immune response [40]. In the MAPK signaling pathway, the BAK1 gene (*CCA0063S0010* and *CCA0507S0134*) in Chinese hickory exhibits significant expression during the early and mid stages. BAK1 can form complexes with other receptor kinases, such as FLS2 (Flagellin-Sensitive 2), to initiate the PTI response. This response includes ion flux, ROS (Reactive Oxygen Species) accumulation, and the activation of the MAPK pathway, as well as the expression of disease resistance genes, thereby enhancing the plant’s ability to resist pathogens [41]. Studies in tobacco(*Nicotiana tabacum*) have shown that BAK1 stabilizes the receptor-like kinase BIR2 by preventing its ubiquitination and degradation, which in turn strengthens the PTI response [42]. Furthermore, research indicates that knocking out the BAK1 negative regulator PP2C15 significantly boosts plant disease resistance [43]. Collectively, these findings suggest that the increased expression of BAK1-related genes may serve as a key indicator of enhanced disease resistance in hickory.

In the plant–pathogen interaction pathway, the fungal hyphal invasion triggers the recognition of the pathogen by resistance genes such as CF9. This recognition activates the PTI response. In Chinese hickory, at various stages of infection, genes downstream of CF9—including CDPK, Rboh, and CaM—are significantly expressed. These genes collectively promote the burst of ROS and hypersensitive responses, which help to inhibit pathogen invasion.

Additionally, plants can prevent further pathogen invasion by reinforcing their cell walls and synthesizing antimicrobial compounds [44]. The phenylpropanoid biosynthesis pathway plays a crucial role in producing antimicrobial agents such as flavonoids, isoflavones, and resveratrol. This pathway is also the primary route for lignin synthesis, which strengthens the cell wall and provides structural defense against pathogens. Three key enzymes—phenylalanine ammonia-lyase (PAL), 4-coumarate-CoA ligase (4CL), and C4H—are critical in regulating the production of major disease-resistant compounds, including lignin, resveratrol, quercetin, catechins, and isoflavones [45]. The expression levels of the related genes in hickory are higher during the early and mid stages of susceptibility, as well as in infected tissue compared to healthy tissue. Similarly, in grafted cucumber(*Cucumis sativus*) studies, genes associated with the phenylpropanoid pathway were found to be significantly up-regulated, resulting in increased lignin content and enhanced disease resistance in the plant [46]. A similar pattern was observed in strawberry (*Fragaria* × *ananassa*) plants infected with *Colletotrichum siamense*, where the “Phenylpropanoid biosynthesis” KEGG pathway was significantly enriched [47]. Notably, the PAL pathway is essential for the synthesis of salicylic acid, a key signaling molecule in plant defense responses.

In the plant hormone signaling pathway, salicylic acid (SA) plays an essential role in defending against biotrophic pathogens during the early stages of infection. The hallmark gene associated with this response is the pathogenesis-related protein PR1, which operates through two main mechanisms. First, as a core regulatory factor in the salicylic acid signaling pathway, PR1 regulates the expression of other disease-resistance genes [48]. Second, as an antimicrobial protein, it directly contributes to the plant’s defense against pathogens. In the infected tissues of walnuts, *PR1*-related genes that are significantly expressed include *CCA0665S0064*, *XLOC_017936*, and *XLOC_001558*. Research on grapevines (*Vitis vinifera*) has also shown that SA can induce the up-regulation of PR1 and related signaling pathway genes, including WRKY1, NPR1, and TGA2 [49].

The secretion of fungal effectors can activate the effector signaling pathway in plant–pathogen interactions, leading to the regulation of specific plant genes. In this pathway, WRKY2 (*CCA0595S0044*) acts as a key transcription factor, which is critical in modulating the host plant’s immune response. When fungal effector proteins are released, they bind to the receptors on the host plant, triggering the activation of WRKY2. Once activated, WRKY2 enhances the expression of pathogenesis-related (PR) genes, thereby boosting the plant’s disease resistance. In addition, WRKY2 contributes to activating antioxidant responses and the salicylic acid (SA) signaling pathway, further fortifying the plant’s defense against pathogen invasion [50,51]. In a study of *Nicotiana attenuata* infected by *Alternaria alternata*, it was found that WRKY family members are key regulatory factors. They activate the expression of defense-related genes by binding to the promoter regions of several genes, thereby enhancing the plant’s disease resistance [52].

In summary, the MAPK signaling pathway, phenylpropanoid biosynthesis pathway, plant hormone signal transduction, and plant–pathogen interaction pathways play crucial roles in Chinese hickory’s defense against pathogen infection. The main defense strategies employed by Chinese hickory include strengthening lignin synthesis, secreting antimicrobial secondary metabolites, synthesizing pathogenesis-related proteins to inhibit pathogen growth, and inducing a hypersensitive response. Key genes involved in these defense mechanisms include receptor kinase BAK1-related genes (*CCA0063S0010* and *CCA0507S0134*), pathogenesis-related protein PR1-related genes (*CCA0665S0064*, *CCA0870S0007*, *CCA1430S0006*, *XLOC_001555*, *XLOC_017937*, *XLOC_017936*, *XLOC_017938* and *XLOC_001558*), transcription factor WRKY2-related gene (*CCA0595S0044*), PAL-related genes (*CCA0589S0228*, *CCA0546S0150*, *CCA1013S0012*, *CCA1591S0016*, *CCA1591S0017*, and *CCA0589S0229*), C4H-related genes (*CCA0905S0011* and *CCA0691S0117*), and 4CL-related genes (*CCA0578S0022*, *CCA0918S0077*, *CCA0918S0075*, *CCA0708S0027*, *CCA0879S0055*, and *CCA0662S0022*). These genes are essential for enhancing Chinese hickory disease resistance and combating pathogen infection.

The interaction between plants and pathogens is a complex process, influenced by various factors such as the extent of pathogen infection, gene regulation, and environmental conditions. Enrichment analysis of differentially expressed genes (DEGs) during different stages of Chinese hickory infection revealed that several common resistance pathways were enriched in the infected tissues during the early and mid stages of infection. These pathways include the MAPK signaling pathway, phenylpropanoid biosynthesis, plant hormone signal transduction, plant–pathogen interactions, flavonoid biosynthesis, and stilbenoid biosynthesis. Notably, some of these pathways were also enriched in the infected tissues of the early and mid-infection stages in relation to lncRNA and circRNA.

The study of differential gene expression between Cc_M_D vs. Cc_P_D and Cc_A_D vs. Cc_M_D revealed significant changes in gene expression within disease-related pathways at different stages of susceptibility. In the mid-susceptible stage, compared to the early stage, the expression of peroxidase genes associated with lignin synthesis was up-regulated. This finding aligns with disease progression in hickory, where the peak of dry rot occurs in April and May, during which the xylem forms black lesions and sustains considerable damage. To counter this, Chinese hickory increases the expression of genes involved in suppressing the infection by *B. dothidea*. Lignin accumulation plays a critical role in enhancing plant disease resistance. In resistant rice (*Oryza sativa*) varieties, the Xa7 gene boosts the lignin biosynthesis pathway, improving resistance to *Xanthomonas oryzae* [53]. Similarly, in wheat (*Triticum aestivum*), mutations in the BGI1 gene reduce lignin content, compromising the wheat’s resistance to *Fusarium* [54].

Furthermore, during the mid-susceptible stage of Chinese hickory, genes associated with peroxidase activity are up-regulated compared to the early stage. Overexpression of peroxidase enhances plant peroxidase activity and antioxidant capacity, thereby improving the plant’s tolerance to oxidative stress induced by reactive oxygen species (ROS) and strengthening its resistance to fungal pathogens [55]. Similar studies have demonstrated that peroxidase genes in sugarcane(*Saccharum officinarum*) are significantly up-regulated in response to *Saccharum mosaic* virus infection [56]. In cotton (*Gossypium hirsutum*), multiple peroxidase genes are significantly activated following infection by *Verticillium dahliae*, underscoring their crucial role in disease defense [57]. Moreover, in *Arabidopsis thaliana*, members of the peroxidase gene family have been shown to regulate ROS levels under both biotic and abiotic stress, thereby contributing to the plant’s overall stress resistance [58]. For instance, in tobacco, high expression of peroxidase-related genes has been shown to confer greater tolerance to methyl viologen-induced oxidative stress, as well as enhanced resistance to the oomycete pathogen *Phytophthora nicotianae*, the causative agent of tobacco black shank disease [59].

Differential gene clustering revealed distinct gene expression patterns in the infected tissues of Chinese hickory during the late infection stage compared to the early and mid stages. This observation is further supported by clustering results of differentially expressed lncRNAs and circRNAs. Heatmap analysis of the Chinese hickory disease-resistance pathways showed that most resistance genes had much higher expression levels in the early and mid stages of infection than in the late stage. Pathogenesis-related protein 1 (PR1), a common marker of plant immune response, exhibited elevated expression in the early and mid stages but was nearly undetectable in the late stage. This suggests that the Chinese hickory’s resistance is weakest during the late infection stage, possibly due to *B. dothidea* evading the plant’s defense mechanisms by that point [60]. In the late stage of disease susceptibility, compared to the mid stage, there is also an up-regulation of certain genes, particularly those involved in the Ca^2+^ signaling pathway, such as calcium-dependent protein kinase (CDPK), respiratory burst oxidase homolog (Rboh), and calmodulin (CAM). These genes promote the burst of reactive oxygen species (ROS), which in turn triggers the hypersensitive response and enhances the plant’s defense against pathogens. The up-regulation of these genes may be an attempt by Chinese hickory to counteract pathogens by increasing ROS production during the later stage of infection. However, due to the significant decrease in the expression of other disease-resistance genes, such as *PR1*, the overall disease resistance of the Chinese hickory remains weak. The gene expression pattern in the later stage of disease susceptibility reveals the complexity of its defense mechanisms: while some genes (e.g., CDPK, Rboh, and CAM) are up-regulated to enhance ROS production, the down-regulation of most disease resistance genes, such as *PR1*, weakens the overall disease resistance.

NPR1 interacts with members of the TGA transcription factor family to form the NPR1-TGA complex. The TGA transcription factor, in turn, binds to the promoter of the salicylic acid-induced gene *PR1*, thereby promoting its expression. This study observed two distinct expression patterns of NPR1 in the interaction between American hickory and *B. dothidea*: NPR1 (*CCA0526S0239*) is predominantly expressed during the mid-to-late stages of infection, while NPR1 (*CCA0526S0240*) is primarily expressed during the early and mid-infection stages. These differences suggest that Chinese hickory may utilize distinct immune strategies to combat pathogen infection at various stages of the disease. During the early and mid-infection stages, the high expression of NPR1 (*CCA0526S0240*) may be associated with early pathogen recognition and rapid stress response. In a study of chili pepper (*Capsicum annuum*), the CaNPR1 gene is primarily expressed early in the disease process, enhancing plant resistance to viral infections by activating salicylic acid (SA)-dependent disease resistance genes [61]. Furthermore, in citrus(*Citrus reticulata*) research on resistance to citrus canker, the NPR1-like gene is rapidly activated in resistant varieties, promoting the SA signaling pathway and enhancing the synthesis of peroxidases (POD) and secondary metabolites [62]. Based on these findings, we hypothesize that NPR1 (*CCA0526S0240*) in the early and mid-infection stages of American hickory may trigger early defense mechanisms through the SA-dependent pathway, improving the plant’s ability to recognize and respond to pathogens. In the mid-to-late stages of infection, NPR1 (*CCA0526S0239*) is up-regulated, which may be associated with systemic acquired resistance (SAR). SAR is a crucial mechanism for enhancing overall immunity in plants during late-stage pathogen infection. In a study of potatoes (*Solanum tuberosum*), StNPR1 is up-regulated in the later stages of the disease, activating PR1, β-1,3-glucanase, and PAL through the SA pathway, thereby enhancing disease resistance [63].

Compared to our laboratory’s previous study on the interaction mechanism between Chinese hickory and *B. dothidea* under controlled indoor conditions [17], both studies revealed significant enrichment of pathways related to secondary metabolite biosynthesis, photosynthesis, and plant hormone signal transduction in the differentially DEGs analysis. In the indoor experiment, we measured changes in SA and JA levels during hickory infection and found that SA levels significantly decreased after *B. dothidea* infection, whereas JA levels exhibited minor fluctuations but increased in the later stages of infection. This finding aligns with the results of the present study: in the early and middle stages of infection, SA-related gene expression was significantly higher in infected tissues than in healthy tissues, while JA-related gene expression remained low during the early and middle stages but increased significantly in the late stage of infection (Figure 5B). However, the two studies showed contrasting trends in DEG numbers: in the indoor experiment, the number of DEGs between infected and healthy tissues continuously increased throughout disease progression, whereas in the present outdoor experiment, the number of DEGs displayed a decreasing trend. We speculate that this difference may be attributed to the duration of the experiments. The indoor experiment was relatively short (15 days), resulting in a more concentrated and intense response of Chinese hickory to the pathogen. In contrast, the outdoor experiment lasted five months, during which the expression of disease resistance-related genes in hickory was gradually suppressed over the prolonged infection period. This extended duration likely provides a more realistic representation of the interaction between hickory and *B. dothidea* under natural conditions.

The findings of this study help elucidate the molecular mechanisms underlying Chinese hickory resistance to *B. dothidea* under outdoor conditions, providing valuable insights for selecting disease-resistant varieties and developing effective disease management strategies. Through transcriptome sequencing and DEG analysis, we identified several key genes involved in the interaction between Chinese hickory and *B. dothidea*. These findings offer critical molecular targets for screening disease-resistant varieties and can be leveraged to develop marker-assisted selection (MAS) techniques, accelerating the breeding of resistant cultivars. Furthermore, gene editing technologies such as CRISPR/Cas9 could be employed to enhance or stabilize the expression of resistance-related genes [64], improving disease resistance and facilitating the development of highly resistant varieties. Additionally, this study highlights the essential role of non-coding RNAs in disease defense, particularly in cell wall reinforcement, redox enzyme regulation, and antimicrobial secondary metabolite biosynthesis. These insights provide a theoretical foundation for RNA-based molecular regulation techniques, such as RNA interference (RNAi). Moreover, we observed significant differences in the expression patterns of resistance genes across different stages of infection, offering scientific guidance for precision agronomic management and optimized biological control strategies. For instance, enhancing disease resistance in Chinese hickory during the early stages of infection through elicitors or plant hormone regulation, combined with targeted chemical treatments at appropriate infection stages, could improve disease control efficacy while reducing reliance on traditional fungicides. Additionally, analyzing gene expression patterns across different Chinese hickory varieties enables the identification and prioritization of cultivars that exhibit sustained high expression of resistance genes in the early and middle stages of infection. In conclusion, this study provides new perspectives and a strong research foundation for selecting disease-resistant Chinese hickory varieties and implementing scientifically informed disease management. It holds significant implications for future research and practical applications in agriculture and horticultural management.

## 4. Materials and Methods

### 4.1. Plant Materials

The plant material of Chinese hickory was sourced from a Chinese hickory plantation located in Wu Village, Taiyang Town, Lin’an District, Hangzhou City, Zhejiang Province, with geographical coordinates of 119°28′ E and 30°31′ N. Three adult hickory trees, similar in age, growth, and health, were selected from the same location and designated as Tree 1, Tree 2, and Tree 3 (Figure 1A). Based on the biological characteristics of *B*. *dothidea*, samples were collected at three stages of dry rot disease infection in Chinese hickory: early-infection (24 March 2022), mid-infection (26 May 2022), and late-infection (5 August 2022) (Figure 1B). Bark samples (≥1.5 g/sample) were taken from both infected and healthy areas using a bark perforator and placed into centrifuge tubes (Figure 1C). These samples were immediately immersed in liquid nitrogen. After being transported under low-temperature conditions to the laboratory, the samples were stored at −80 °C for subsequent analysis. At the same time, observations and recordings focused on the condition of the bark at the diseased sites during three stages: early infection, mid-infection, and late infection, with healthy bark serving as the control. The trees included in the experiment were all naturally infected. Trees 1, 2, and 3 were spaced 10–20 m apart. For each tree, infected bark samples from diseased areas were collected at three different time points from nearby locations, following the same approach for healthy bark samples. The sampling points for infected and healthy bark on each tree were approximately 20 cm apart.

### 4.2. Laboratory Reagents

Transcriptome sequencing of both infected and normal tissues of Chinese hickory was conducted by Beijing Novogene Science and Technology Co., Ltd. (Beijing, China). DEPC water for RNA extraction was obtained from Dingguo Changsheng Co., Ltd. (Beijing, China), while β-mercaptoethanol was sourced from Shenggong Biotechnology Co., Ltd. (Shanghai, China). Chloroform, isoamyl alcohol, and isopropanol were purchased from Xilong Chemical Co., Ltd. (Beijing, China). Water-saturated phenol (250 mL) was provided by Shanghai Kanglang Technology Co., Ltd. (Shanghai, China). DNase I, agarose gel electrophoresis markers, and the reverse transcription reagent kit HiScriptIIQ RT SuperMix were acquired from Tiangen Biochemical Technology (Beijing) Co., Ltd. (Beijing, China). 

### 4.3. Chinese Hickory Transcriptome Sequencing

Transcriptome sequencing was performed on Chinese hickory samples collected at three stages of infection (early-infection, mid-infection, and late-infection) outdoors. Infected tissues from each stage were labeled as Cc_P_D (early), Cc_M_D (middle), and Cc_A_D (late), while healthy tissues at the same time points as the controls were designated as Cc_P_W (early), Cc_M_W (middle), and Cc_A_W (late). Each treatment included three biological replicates.

The samples were ground into a fine powder using liquid nitrogen and transferred to 2.0 mL EP tubes containing 2% CTAB lysis buffer. The mixture was then vortexed thoroughly and incubated at 65 °C for 20 min. After incubation, the supernatant was collected by centrifugation for 5 min. A chloroform-isoamyl alcohol mixture (24:1) was added and shaken vigorously to mix. The supernatant was separated after centrifugation for 10 min.

Next, the supernatant was transferred to a mixture of phenol, chloroform, and isoamyl alcohol (25:24:1) and inverted several times to mix. After centrifugation for 10 min, the supernatant was collected. These steps (adding chloroform/isoamyl alcohol and phenol/chloroform/isoamyl alcohol mixtures) were repeated once to further purify the RNA. The chloroform/isoamyl alcohol mixture (24:1) was added again, and the mixture was vigorously inverted. The supernatant was separated by centrifugation for 10 min.

The liquid was then transferred to a new 1.5 mL centrifuge tube, and isopropanol was added. The solution was placed at −20 °C for 1 h, followed by centrifugation for 20 min. The supernatant was discarded, and the RNA pellet was washed with 75% ethanol. Finally, the RNA was dissolved in DEPC-treated water. The purity and integrity of the RNA were evaluated using 1.5% agarose gel electrophoresis, a NanoDrop 8000 spectrophotometer, and a Bioanalyzer 2100 system. To meet the required standards, the minimum RNA Integrity Number (RIN) threshold was set to 7.

The sequencing libraries were constructed according to Illumina’s standard protocol for library preparation and sequencing [65]. Separate libraries were prepared for transcriptome sequencing, lncRNA sequencing, and circRNA sequencing. After passing quality control tests, the libraries were pooled based on the required effective concentration and target data volume. The pooled libraries were then sequenced using the Illumina NovaSeq 6000 platform, generating 150 bp paired-end reads.

### 4.4. Quality Control of Chinese Hickory Transcriptome Data

Fastp (v0.23.4, OpenGene) was used to filter the raw sequencing data by removing reads with adapter sequences, reads containing ‘N’ (indicating undetermined base calls), and low-quality reads (defined as those where more than 50% of the bases have a Phred quality score ≤ 20). The clean data were then analyzed to calculate the Q20, Q30, and GC content. All subsequent analyses were performed based on the clean data.

### 4.5. Transcriptomic Data Analysis of Chinese Hickory

The Chinese hickory reference genome and genome annotation files were downloaded from NCBI (https://www.ncbi.nlm.nih.gov/assembly/GCA_011037825.1/, accessed on 5 December 2022) [66]. The HISAT2 software (v2.2.1, Johns Hopkins University) was used to run the hisat2-build command to construct an index for the Chinese hickory reference genome. The hisat2 -x -1 -2 -S.bam command was then used to align paired-end clean reads from different Chinese hickory samples to the reference genome, obtaining the mapping information of the sequencing reads on the genome. The alignment results in BAM format was converted to SAM format using Samtools (v1.17, Sanger Institute). Subsequently, StringTie (v1.3.3b, Johns Hopkins University) was used to assemble the alignment results. The alignment files (BAM) and other parameters, such as the -f option specifying the GTF annotation file for each sample and the -G option for the reference genome’s GTF annotation, were set. Transcripts with uncertain strand orientation or lengths less than 200nt were removed. The remaining transcripts were compared with known databases, and known transcripts were filtered out. Finally, the new transcripts were subjected to coding potential prediction, resulting in the identification of Novel_lncRNA and Novel_mRNA.

The expression levels of known transcripts, as well as the predicted novel lncRNAs, novel mRNAs, and unclassified transcripts, were quantified. Their FPKM (Fragments Per Kilobase per Million mapped reads) values were calculated for further analysis. CircRNAs were detected and identified using both the find_circ (v1.2, Johns Hopkins University) [67] and CIRI2 (v2.0.5, Shenzhen Institutes of Advanced Technology) tools [68], and their expression levels were calculated and normalized using TPM (Transcripts Per Million) [69]. Functional annotation of the Chinese hickory genes was performed using the eggNOG-mapper database (http://eggnog6.embl.de/, accessed on 28 December 2022).

### 4.6. Differential Gene Expression Analysis of Chinese Hickory

Differentially expressed genes (DEGs) were identified using DESeq2 (v1.20.0, part of the Bioconductor project) [70] for whole-transcriptome analysis, with significance thresholds set at padj < 0.05 and |log2(fold change)| ≥ 1. The union of DEGs from all comparison groups was taken as the final DEG set. For lncRNA differential expression analysis between two comparison groups, edgeR (v3.22.5) was used, with *p*-values adjusted using the Benjamini and Hochberg method. Genes with an adjusted *p*-value < 0.05 were considered significantly differentially expressed. Similarly, circRNA differential expression analysis was performed using DESeq2 (v1.20.0, part of the Bioconductor project), applying the same *p*-value adjustment method, with circRNAs meeting the adjusted *p*-value < 0.05 threshold classified as differentially expressed. Cluster analysis was conducted separately for DEGs in the whole transcriptome, lncRNAs, and circRNAs. Additionally, GO functional enrichment and KEGG pathway enrichment analyses were performed on the DEG sets of the whole transcriptome, lncRNAs, and circRNAs using clusterProfiler (v3.8.1, Guangzhou Institutes of Biomedicine and Health) [71].

### 4.7. Alternative Splicing Analysis

Alternative splicing analysis was performed using rMATS software (version 4.0.2, University of Southern California), which generates a list of differential splicing events. To assess the significance of these events, two main criteria were applied: False Discovery Rate (FDR) and deltaPSI (differential Percent Spliced In). The absolute value of deltaPSI was set to 0.1, and the FDR threshold was set at 0.05. These parameters were used to identify significant splicing events for further analysis.

## 5. Conclusions

This study conducted transcriptome sequencing on infected and healthy tissues of Chinese hickory at different stages of infection under outdoor conditions. DEGs analysis was performed on the sequencing data to identify key pathways and genes involved in the plant’s defense response, providing a systematic exploration of the transcriptomic response of Chinese hickory to *B*. *dothidea*.

Our findings revealed that in the early and middle stages of infection, genes associated with lignin biosynthesis, peroxidase activity, and plant hormone signaling exhibited significant differential expression in infected tissues compared to healthy tissues. Additionally, an analysis of lncRNAs and circRNAs in outdoor Chinese hickory samples at different infection stages showed that non-coding RNAs also contribute to pathogen defense by reinforcing cell walls, modulating redox enzyme activity, and synthesizing secondary metabolites with antibiotic properties.

Furthermore, we observed distinct gene expression patterns between early/middle-stage and late-stage infected tissues. In the late stage of infection, the expression of most genes involved in resistance pathways was suppressed, with key defense-related genes such as PR1 and WRKY2 showing a downward trend. These findings provide new insights and a strong research foundation for identifying disease-resistant Chinese hickory varieties and developing effective management strategies for dry rot disease, offering valuable guidance for future research and practical applications.

## Data Availability

Raw Illumina reads have been deposited at the NCBI Sequence Read Archive (BioProject: PRJNA1199706, https://dataview.ncbi.nlm.nih.gov/object/PRJNA1199706?reviewer=qvlkt8hfj1qgkdp10ketun7msa, accessed on 29 December 2024).

## Supplementary Materials

The following supporting information can be downloaded at: https://www.mdpi.com/article/10.3390/plants14050793/s1.

## Abbreviations

The following abbreviations are used in this manuscript:

PRR	Pattern Recognition Receptor
PAMPs	Pathogen-Associated Molecular Patterns
PTI	PAMP-Triggered Immunity
ETS	Effector-Triggered Susceptibility
ETI	Effector-Triggered Immunity
DEG	Differentially Expressed Gene
GO	Gene Ontology
KEGG	Kyoto Encyclopedia of Genes and Genomes
FDR	False Discovery Rate
deltaPSI	differential Percent Spliced In
NPR1	Non-expressor of Pathogenesis-related Gene 1
MAPK	Mitogen-Activated Protein Kinase
ROS	Reactive Oxygen Species
Chi B	Basic endochitinase B
PAL	Phenylalanine Ammonia-Lyase
4CL	4-coumarate-CoA Ligase
C4H	Cinnamate-4-hydroxylase
SA	Salicylic Acid
ICS	Isochorismate Synthase
JA	Jasmonic Acid
JAZ	Jasmonate Zim Domain
PR	Pathogenesis-Related Protein
CDPK	Calcium-Dependent Protein Kinase
Rboh	Respiratory Burst Oxidase
CAM	Calmodulin
HR	Hypersensitive Response
SE	Skipped Exon
RI	Retained Intron
MXE	Mutually Exclusive Exon
FLS2	Flagellin-Sensitive 2
MAS	marker-assisted selection
RIN	RNA Integrity Number
