# Supplementary material for: Mechanisms of Chinese Hickory Resistance to Dry Rot Disease by Botryosphaeria dothidea: A Comprehensive Analysis from Gene Expression to Non-Coding RNAs
Source: Plants (Basel). 2025 Mar 4;14(5):793. doi: 10.3390/plants14050793 (PMC11901809; doi:10.3390/plants14050793)
Supplement: Supplementary file 1 [file plants-14-00793-s001.zip › e-Xtras.pdf]

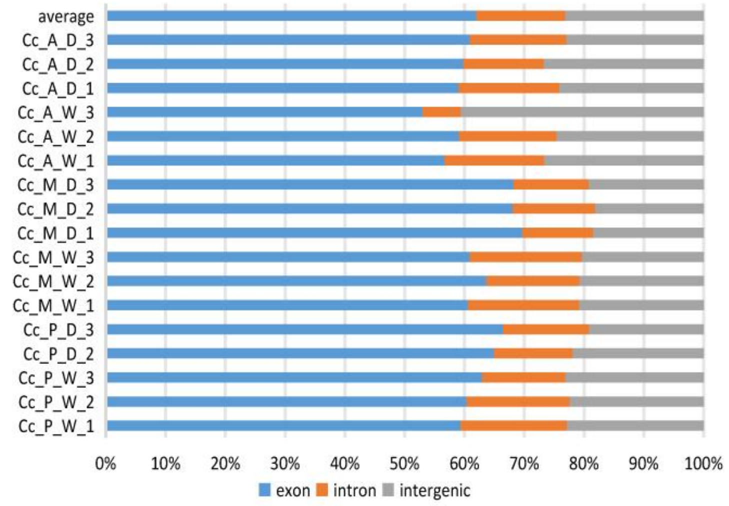

Figure S1. The sequencing data alignment results for Chinese hickory.Distribution of genomic regions in different samples: blue represents exons, orange represents introns, and gray represents intergenic regions.

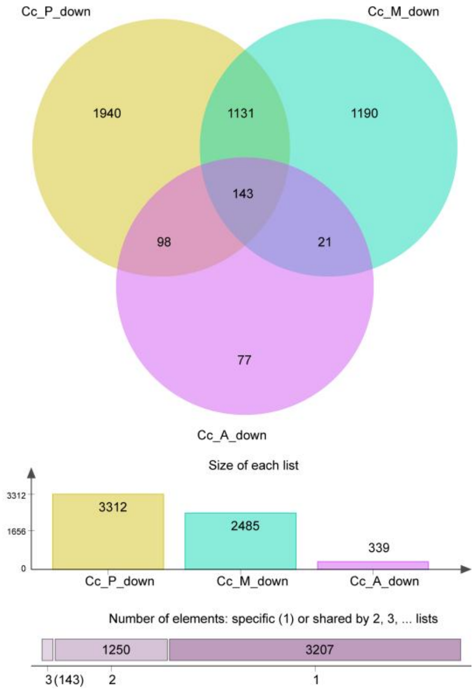

Figure S2.The Venn diagram illustrates down-regulated DEGs across different comparison groups. Cc_P_down refers to the comparison between healthy tissue and infected tissue during the early-infection, Cc_M_down represents the comparison in the mid-infection, and Cc_A_down refers to the comparison in the late-infection. This diagram highlights both the number of down-regulated DEGs unique to each comparison group and the number of shared down-regulated DEGs. The yellow, blue, and purple bar charts display the total number of down-regulated DEGs for the early, middle, and late--infection, respectively, comparing healthy

tissue to infected tissue. The stacked bar chart, from left to right, shows the number of down-regulated DEGs shared by all three groups, shared by two groups, and uniquely found in one group.

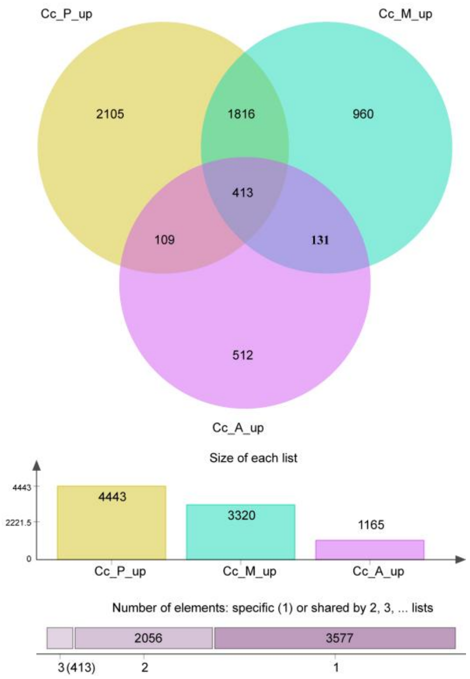

Figure S3.The Venn diagram illustrates up-regulated DEGs across different comparison groups. Cc_P_up refers to the comparison between healthy tissue and infected tissue during the early-infection, Cc_M_up represents the comparison in the mid-infection, and Cc_A_up refers to the comparison in the late-infection. This diagram highlights both the number of up-regulated DEGs unique to each comparison group and the number of shared up-regulated DEGs. The yellow, blue, and purple bar charts display the total number of dup-regulated DEGs for the early, middle, and late-infection, respectively, comparing healthy tissue to infected tissue. The stacked bar chart, from left to right, shows the number of up-regulated DEGs shared by all three groups, shared by two groups, and uniquely found in one group.

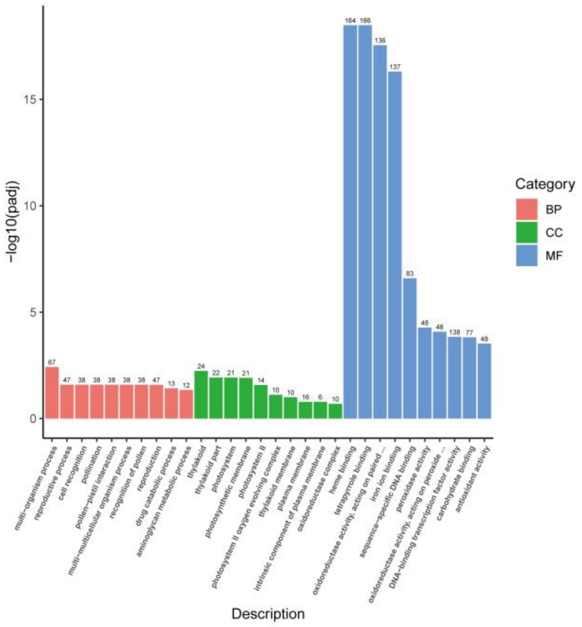

Figure S4. GO enrichment analysis in Cc_M_D *vs* Cc_M_W. The GO terms significantly enriched in the diseased middle-stage infected tissues compared to healthy tissues were analyzed. BP, CC, and MF refer to the biological process, cellular component, and molecular function GO terms, respectively, that were significantly enriched.

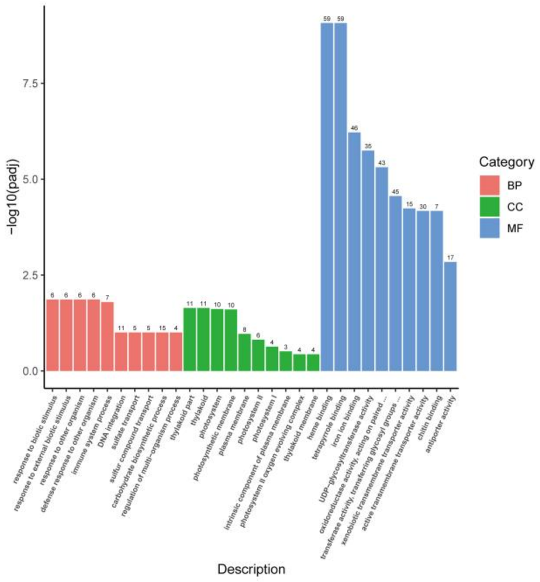

Figure S5. GO enrichment analysis in Cc_A_D *vs* Cc_A_W. The GO terms significantly enriched in diseased late-stage infected tissues compared to healthy tissues were analyzed. BP, CC, and MF refer to the biological process, cellular component, and molecular function GO terms,

respectively, that were significantly enriched.

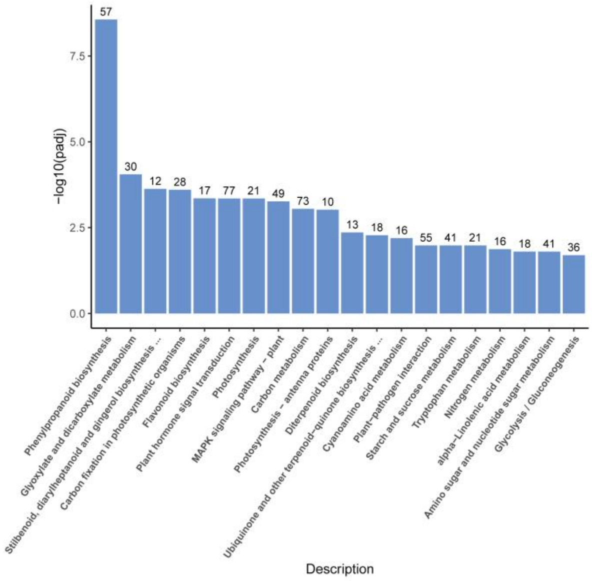

Figure S6. KEGG enrichment analysis in Cc_M_D *vs* Cc_M_W. The KEGG pathways significantly enriched between diseased middle-stage infected tissues and healthy tissues were compared.

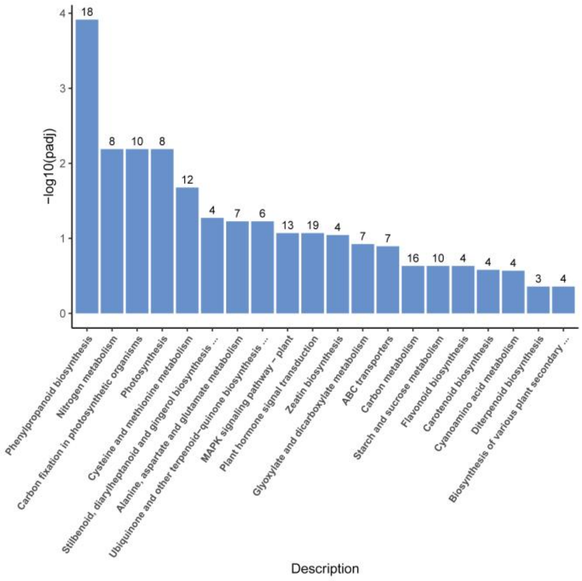

Figure S7. KEGG enrichment analysis in Cc_A_D *vs* Cc_A_W. The KEGG

pathways significantly enriched between diseased late-stage infected tissues and healthy tissues were compared.

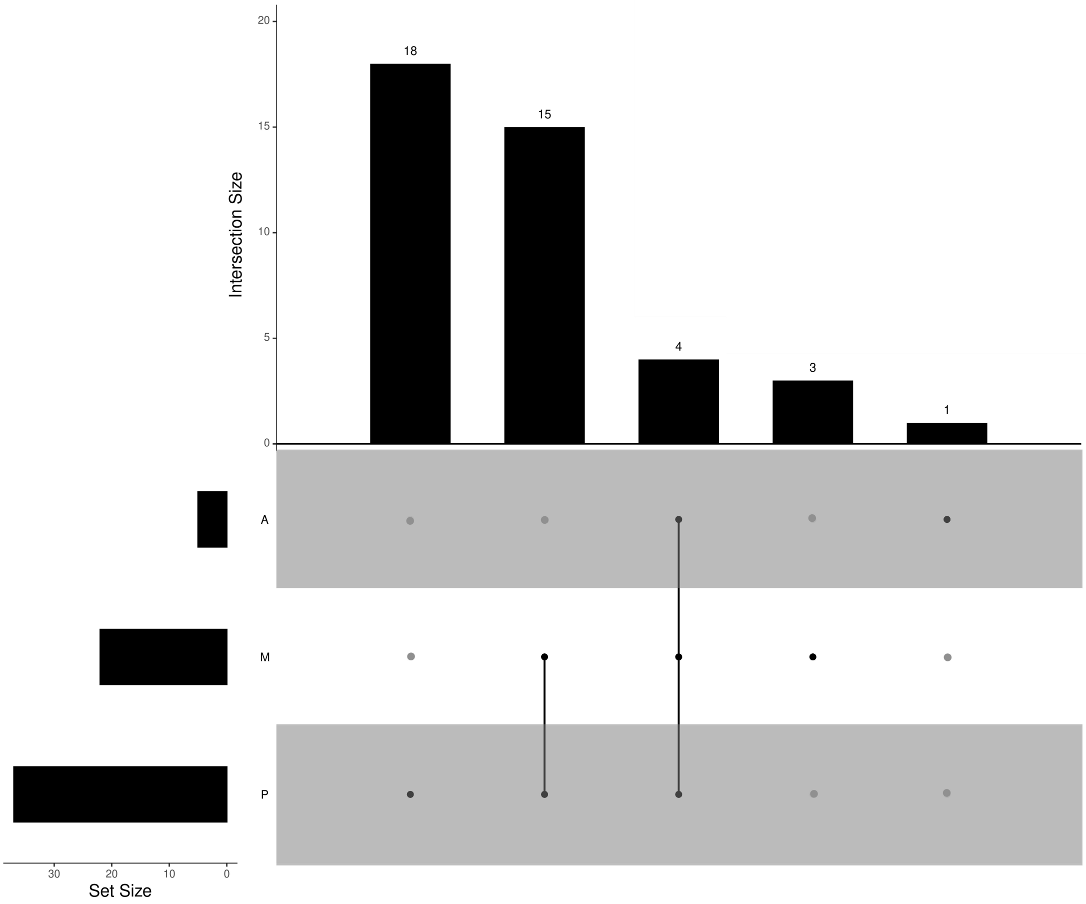

Figure S8. UpSet diagram showing the significantly enriched pathways between different comparison groups. A, M, and P refer to the late-infection, mid-infection, and early-infection.

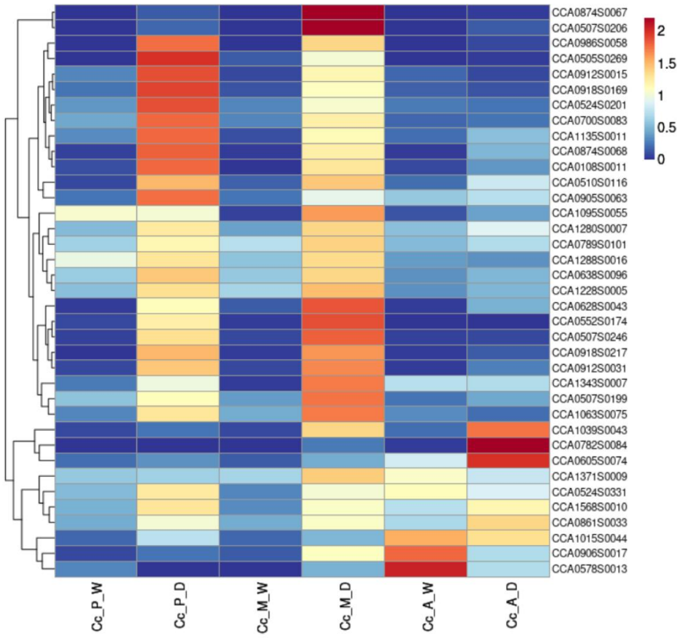

Figure S9. Gene expression profiles of disease-resistant pathways: plant-pathogen interaction. Each row corresponds to a DEG, while each column represents a sample.

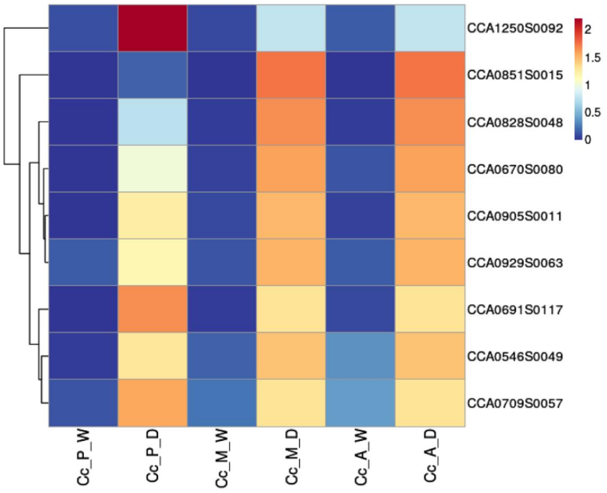

Figure S10.Gene expression profiles of disease-resistant pathways: flavonoid biosynthesis. Each row corresponds to a DEG, while each column represents a sample.

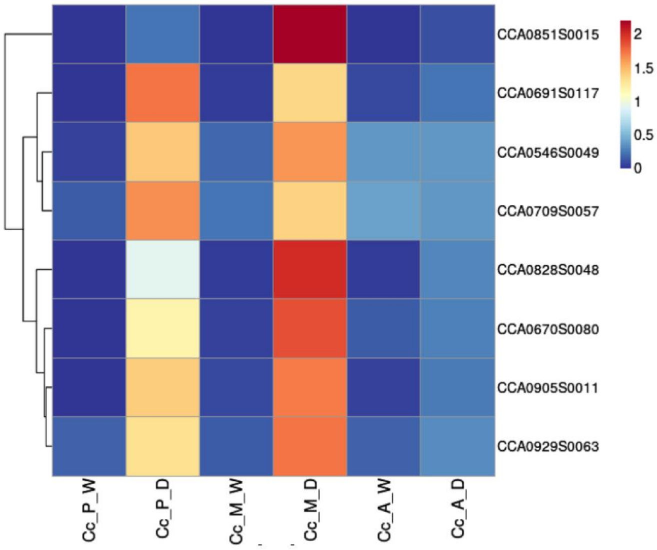

Figure S11. Gene expression profiles of disease-resistant pathways: stilbenoid, diarylheptanoid, and gingerol biosynthesis. Each row corresponds to a DEG, while each column represents a sample.

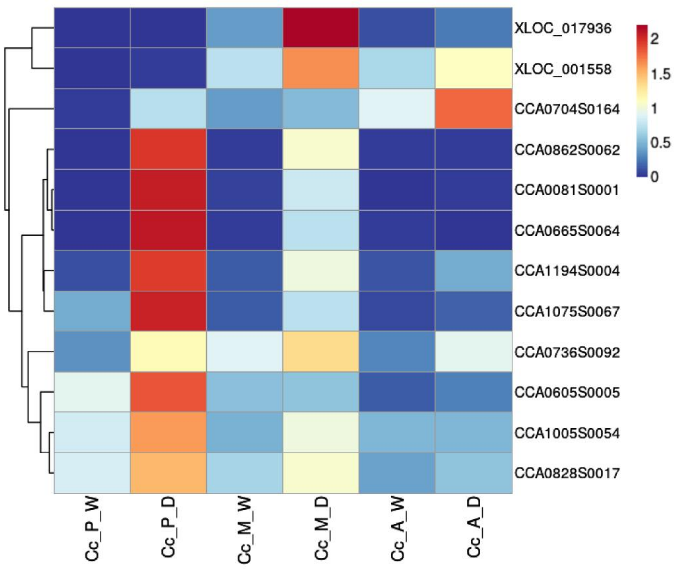

Figure S12. Gene expression profiles of disease-resistant pathways: plant hormone signal transduction. Each row corresponds to a DEG, while each column represents a sample.

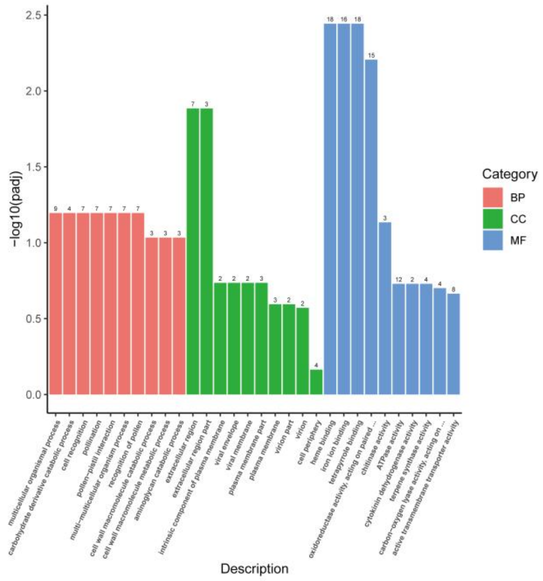

Figure S13. GO enrichment analysis of down-regulated genes in Cc_M_D *vs* Cc_P_D. The GO enrichment terms for down-regulated genes in diseased middle-stage infected tissues compared to diseased early-stage infected tissues were analyzed. BP, CC, and MF refer to the biological process, cellular component, and molecular function GO terms, respectively, that were significantly enriched.

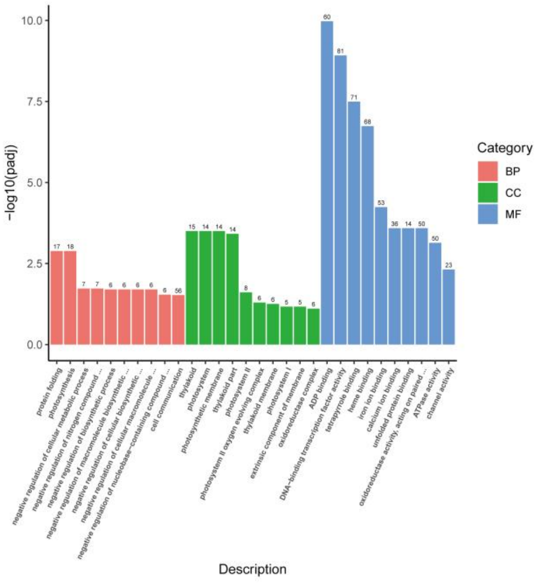

Figure S14. GO enrichment analysis of up-regulated genes in Cc_A_D *vs* Cc_M_D. The GO enrichment terms for up-regulated genes in diseased late-stage infected tissues compared to diseased middle-stage infected tissues were analyzed. BP, CC, and MF refer to the biological process, cellular component, and molecular function GO terms, respectively, that were significantly enriched.

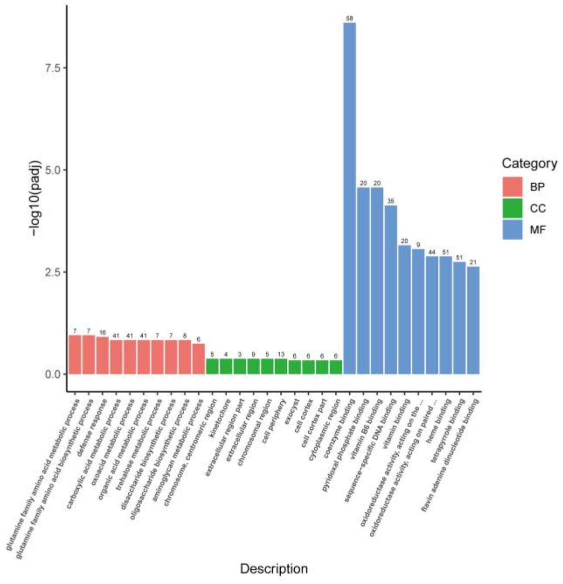

Figure S15. GO enrichment analysis of down-regulated genes in Cc_A_D *vs* Cc_M_D. The GO enrichment terms for down-regulated genes in diseased late-stage infected tissues compared to diseased middle-stage infected tissues were analyzed. BP, CC, and MF refer to the biological process, cellular component, and molecular function GO terms, respectively, that were significantly enriched.

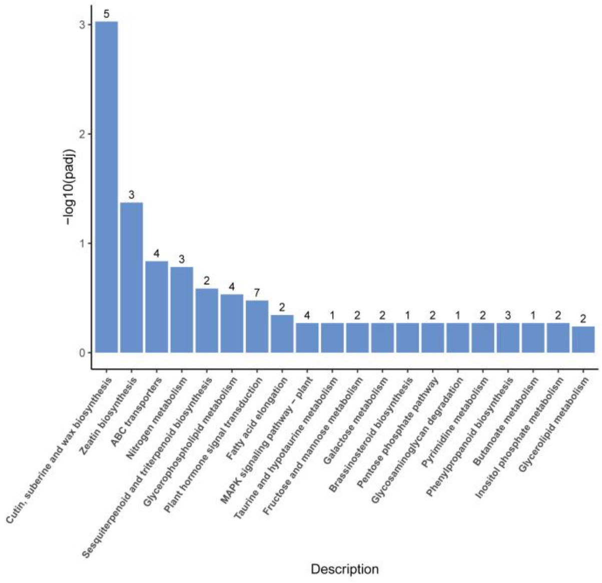

Figure S16. KEGG enrichment analysis of down-regulated genes in Cc_M_D *vs* Cc_P_D. The KEGG enrichment pathways of down-regulated genes in diseased middle-stage infected tissues were compared with those in diseased early-stage infected tissues.

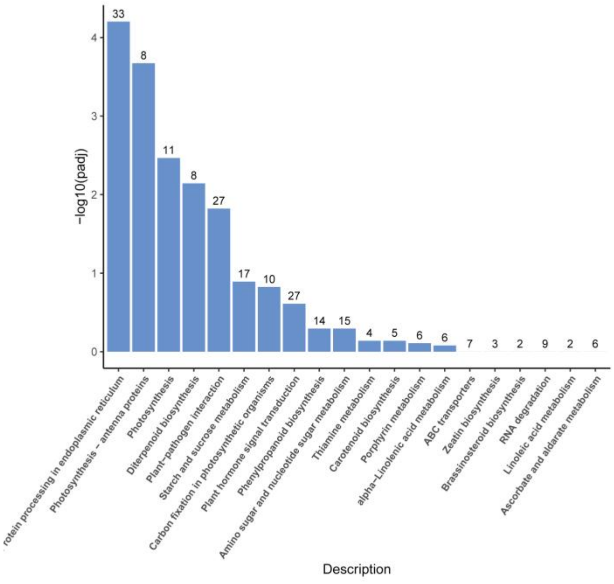

Figure S17. KEGG enrichment analysis of up-regulated genes in Cc_A_D *vs* Cc_M_D. The KEGG enrichment pathways of up-regulated genes in diseased late-stage infected tissues were compared with those in diseased middle-stage infected tissues.

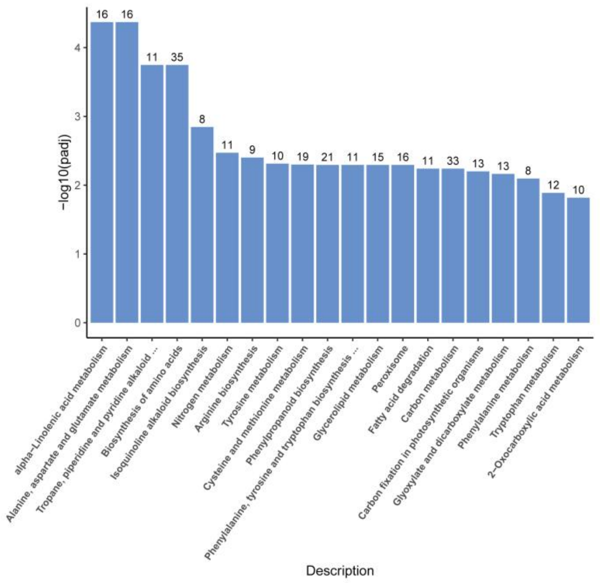

Figure S18. KEGG enrichment analysis of down-regulated genes in Cc_A_D *vs* Cc_M_D. The KEGG enrichment pathways of down-regulated genes in diseased late-stage infected tissues were compared with those in diseased middle-stage infected tissues.
